# The protein and miRNA profile of plasma extracellular vesicles (EVs) can distinguish feline mammary adenocarcinoma patients from healthy feline controls

**DOI:** 10.1038/s41598-023-36110-7

**Published:** 2023-06-06

**Authors:** Jane Howard, John Browne, Stephanie Bollard, Susan Peters, Ciara Sweeney, Kieran Wynne, Shirley Potter, Amanda McCann, Pamela Kelly

**Affiliations:** 1 UCD School of Medicine, College of Health, and Agricultural Sciences (CHAS), University College Dublin, Belfield, Dublin 4, Ireland; 2 UCD Conway Institute of Biomolecular and Biomedical Research, University College Dublin, Belfield, Dublin 4, Ireland; 3 UCD School of Agriculture and Food Science, University College Dublin, Dublin 4, Ireland; 4 Department of Plastic and Reconstructive Surgery, Mater Misericordiae University Hospital, Eccles Street, Dublin 7, Ireland; 5 College of Health and Agricultural Sciences, UCD School of Veterinary Medicine, University College Dublin, Belfield, Dublin 4, Ireland; 6 Systems Biology Ireland, University College Dublin, Belfield, Dublin 4, Ireland

**Keywords:** Breast cancer, Cancer models

## Abstract

Feline mammary adenocarcinomas (FMA) are aggressive tumours with metastatic capability and limited treatment options. This study aims to investigate whether miRNAs associated with FMA tumours are secreted in extracellular vesicles (EVs) and whether they can potentially be used as a cancer biomarker in EVs from feline plasma. Tumours and matched tumour free margins from 10 felines with FMA were selected. Following a detailed literature search, RT-qPCR analyses of 90 miRNAs identified 8 miRNAs of interest for further investigation. Tumour tissue, margins and plasma were subsequently collected from a further 10 felines with FMA. EVs were isolated from the plasma. RT-qPCR expression analyses of the 8 miRNAs of interest were carried out in tumour tissue, margins, FMA EVs and control EVs. Additionally, proteomic analysis of both control and FMA plasma derived EVs was undertaken. RT-qPCR revealed significantly increased miR-20a and miR-15b in tumours compared to margins. A significant decrease in miR-15b and miR-20a was detected in EVs from FMAs compared to healthy feline EVs. The proteomic content of EVs distinguished FMAs from controls, with the protein targets of miR-20a and miR-15b also displaying lower levels in the EVs from patients with FMA. This study has demonstrated that miRNAs are readily detectable in both the tissue and plasma derived EVs from patients with FMA. These miRNAs and their protein targets are a detectable panel of markers in circulating plasma EVs that may inform future diagnostic tests for FMA in a non-invasive manner. Moreover, the clinical relevance of miR-20a and miR-15b warrants further investigation.

## Introduction

Feline mammary tumours are the third most common cancer in domestic cats^1^, with 9 out of 10 mammary tumours being described as malignant^2^. These common tumours primarily occur in older felines between the ages of 10–14^1,2^. Like human breast cancers, feline mammary carcinomas are spontaneous, locally invasive, and often metastatic^3,4^. Feline mammary adenocarcinoma (FMA) is the predominant tumour type reported^2,3^. Bilateral mastectomy remains the most common treatment choice for FMAs and is aims to improve clinical outcomes by reducing recurrence rate and metastatic risk^5^. The benefits of using chemotherapy to treat FMAs are met with conflicting evidence^5–8^, therefore are yet to be implemented as mainstream treatment regimens for felines with mammary tumours. As a result, the prognosis for felines with FMA is poor with the average survival time following diagnosis being 12.3 months^2^. Additionally, studies have shown that up to 67% of FMAs are diagnosed at the metastatic stage^2^ with most cancer progressing to the lymph nodes, lungs, pleura and liver^9,10^. Parameters used in FMA prognoses include tumour size, histological grade, and lymphatic invasion^11^. While these parameters successfully identify patients who are likely to benefit from surgery, they do little to improve patient outcomes.

In human biology, there is a drive to develop molecular biomarkers of disease^12^. More specifically, researchers are aiming to successfully diagnose cancer at earlier stages^13^ using non-invasive, liquid biopsy approaches. In veterinary medicine, there has been significantly less research into the development of such biomarkers, particularly for felines^1,14,15^. As FMAs represent a group of patients with aggressive, potentially metastatic cancer and as such face poor prognosis, the lack of targeted treatment availability highlights FMAs as a disease that may benefit from earlier diagnosis and better prognostic markers. Additionally, the similarities between breast cancers in humans and felines^1,16^ highlights the potential benefit of comparative studies for both patient groups. In human breast cancer, extracellular vesicles (EVs) are gaining traction, not only as potential mediators of breast cancer progression^17–19^ and metastasis^20^ but also as potential biomarkers of disease^21,22^. Specifically, biomarkers from readily available sources such as plasma are being studied in detail^23,24^ and thus highlight their potential importance as easily accessible cancer biomarkers.

Extensive research have detected microRNAs (miRNAs) and detailed their roles in human biology^25^. In human breast cancer, the expression levels of several miRNAs are significantly different between normal and cancerous tissues^26^. Despite significant progress in miRNA biology, only a single study has been published to characterise the feline miRNAome^27^. Furthermore, EVs in the systemic circulation carry cargoes such as miRNA and protein which serve as potential biomarkers for disease monitoring^22,28^. The primary aim of this study is to develop biomarkers for feline mammary adenocarcinoma (FMA). Specifically, we are investigating differences in miRNA expression levels between FMA tumours and the surrounding tumour margins in order to identify potential biomarkers of the disease. Additionally, we aim to expand this biomarker study by investigating the potential of non-invasive circulating extracellular vesicles (EVs) and their cargo as biomarkers. By analysing the levels of circulating EVs and their protein and miRNA cargo components, we hope to determine whether changes in these EVs may reflect the tumour of origin in FMA patients. In addition to their potential for diagnosing and monitoring FMA, these biomarkers may also have the potential to serve as comparative models for studying human diseases.

A detailed literature search using PubMed identified miRNAs with biological significance in human breast cancer. These specific miRNAs were then cross-referenced with the feline miRNAome^27^ and a custom PCR panel was generated using feline-specific assays. To identify specific miRNAs holding biological relevance in feline mammary adenocarcinoma, an initial Cohort of tumours (*Cohort 1*) and matched tumour margins from 10 felines with FMA were selected. An initial screening using RT-qPCR analysis of the 90 miRNAs identified 8 miRNAs of interest for further investigation (Supplementary Information, Table 2). Formalin fixed paraffin embedded tumour tissue (FFPE) and plasma samples were collected from 10 patients (*Cohort 2*) with a diagnosis of FMA (Table 1). Control plasma was also collected from 10 healthy felines undergoing routine elective veterinary procedures that required pre-anaesthetic blood sampling. EVs were isolated from the collected plasma samples and characterised according to the MISEV2018 guidelines^29^. RT-qPCR analysis to examine the expression of the 8 miRNAs of interest was carried out on tumour tissue, tumour margins and plasma EVs. Additionally, proteomic analyses of both control and FMA plasma derived EVs were undertaken.

This study demonstrates the differential expression of miRNAs in tumour tissue compared to tumour margins. Additionally, RT-qPCR demonstrated that circulating plasma EVs reflect the miRNA signature of FMAs. Finally, proteomic analysis of the EVs from felines with FMA, exemplifies a protein footprint that can distinguish those felines with mammary adenocarcinoma from controls. Such plasma biomarkers offer a unique opportunity for veterinarians to screen felines in a relatively non-invasive manner. Together, the miRNA and proteomic signature of circulating plasma EVs present an easily accessible panel of molecular markers detectable using a liquid biopsy. These markers have potential as screening tools to aid the early detection of feline mammary adenocarcinoma.

## Materials and methods

All methods were carried out per relevant guidelines and regulations. Specifically, this study was approved by the Animal Research Ethics Committee (AREC) at University College Dublin (Reference Number: AREC-19-45-Kelly) and written informed consent was obtained from all pet owners upon recruitment.

### Patient recruitment and sample collection

**Figure 1:**
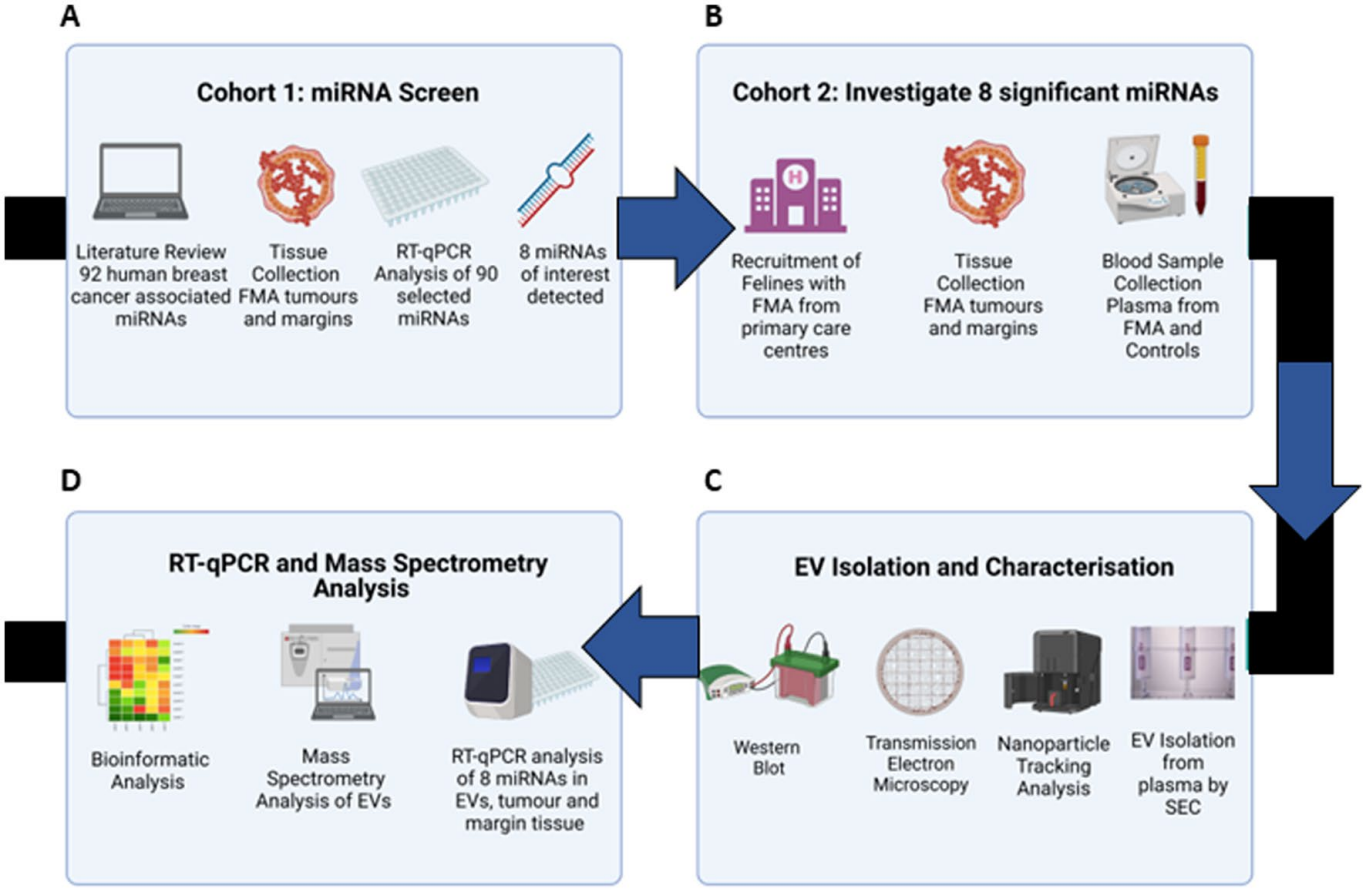
Overview of the experimental design and data analysis pipeline.

Following diagnostic testing, excess tissue samples (FFPE) from patients with FMA were accessed (*Cohort 1*). Suitable tumour samples were selected and graded by a European specialist in Veterinary Pathology (EBVS®) (PK)^11^. Tissue samples included 10 FMA tumour tissue samples and 10 matched tumour margins. The second cohort of patient samples *(Cohort 2)* included 10 FMA tumour tissue samples, their 10 matched tumour margins together with a plasma sample (n = 10) that was collected at the time of mastectomy. Control samples (n = 10) were collected from healthy, female adult cats with no history of mammary disease undergoing routine veterinary procedures that required pre-anaesthetic blood sampling from primary veterinary practices. The study design is summarised in Fig. 1.

### Tissue sample preparation and deparaffinisation

To prepare tissue for RT-qPCR for *Cohort 1 and Cohort 2,* scrolls from both tumour and margin tissue samples were cut to a thickness of 3 µm, and stored in nuclease-free microcentrifuge tubes. For each FFPE tissue sample, a new microtome blade was used, and the stage was cleaned, taking care to minimise RNA contamination between sections. Sections were then stored at −80 °C for subsequent RNA isolation. Briefly, deparaffinisation was carried out using organic solvents; 1 ml of xylene was added to each tissue sample and vortex thoroughly. Samples were then spun at 10,000 × g for 5 min. The xylene was removed. This xylene step was repeated to ensure complete paraffin removal. One ml of 100% ethanol was then added, and samples were spun at 10,000 × g for 5 min. The supernatant was completely removed, and tissue samples were allowed to dry at room temperature for 15 min.

### Plasma sample preparation

Plasma samples were taken from patients with FMA as part of the routine veterinary care in primary veterinary practices. Control plasma was collected from healthy feline patients before they underwent routine elective veterinary procedures. Owner consent was obtained, and all experiments were performed per the protocol approved by the Animal Research Ethics Committee, UCD (Approval ID: AREC-E-19-45-Kelly). All plasma samples were accessed only after all diagnostic tests required by their treating veterinarian were complete. Feline samples were spun at 1900 × g for 15 min to separate the plasma and subsequently spun at 2500 × g for 10 min to remove platelets. Plasma was then stored in 500 µl aliquots and stored at −80 °C for EV isolation.

We note the importance of providing enough information to demonstrate the findings' reliability and robustness, allowing others to replicate and build upon our study. However, in this instance, the ARRIVE guidelines (e.g. animal care and monitoring, housing, and husbandry), are not appropriate for this study, as it did not involve an *in-vivo* experiment. Rather, these animals are pets remained in the care of their owners throughout, and as mentioned samples were only used following diagnostic tests. All experiments were performed per relevant guidelines and regulations.

### Isolation of EVs by size exclusion chromatography

EVs were isolated from plasma by size exclusion chromatography (SEC), using IZON qEV original columns (iZon Science), according to previously published methods^30^. Briefly, the columns were removed from 4 °C to room temperature the 20% ethanol storage solution was removed, and the column flushed with 10 ml Phosphate Buffered Saline (PBS). A 500 µl aliquot of plasma was taken from −80 °C storage, thawed quickly and added to the column. Fractions 7–10, each containing 500 µl of elute were collected according to our previously optimised study^30^. To limit the number of freeze–thaw cycles for each patient plasma sample, fractions 7–10 were pooled, aliquoted, and stored at −80 °C for downstream analyses. EV aliquots for downstream analyses of each sample were as follows; 1 ml were used for RNA analysis, 500 µl for mass spectrometry analysis, 50 µl for BCA protein quantification, 40 µl for NTA. Additionally, the remaining samples were used for TEM and western blot analyses to satisfy the MISEV guidelines^29,30^. There was no residual sample as all EVs isolated from each biological sample were used for analyses.

### miRNA isolation of FFPE samples

Total RNA was extracted from one FFPE tissue slice (each 3 µm thick) using the miRNeasy FFPE Kit (Qiagen, 217.504). Proteinase K Digestion buffer (buffer PKD) and 1 µl RNA isolation spike-in control mix (Qiagen, 339,390) was added. Samples were incubated at 56 °C for 40 min, followed by 80 °C for 15 min. DNase I digestion was carried out, and further steps were performed according to the manufacturer’s protocol. Finally, dried RNA, including miRNA was eluted in 20 µl RNase-free water. Initially, RNA concentration, purity and contamination were quantified using the Nanodrop One spectrophotometer. Following dilution, the RNA concentration of FFPE samples was measured using the Qubit RNA HS Assay Kit on a Qubit 2.0 Fluorometer.

### miRNA isolation of EV samples

Following SEC isolation, EVs from 1 ml of elute were concentrated using Amicon Ultrafiltration 10 kDa filters. Total RNA, including miRNA, was isolated from the EV samples using the miRNeasy micro kit (Qiagen, 217.084) according to the manufacturer’s protocol. Following the addition of Qiazol, 1 µl of carrier RNA and 1 µl RNA isolation spike-in control mix were added. Additionally, the chloroform addition step was repeated twice to ensure pure RNA yield. Finally, dried RNA, including miRNA was eluted in 15 µl RNase-free water. The purity of RNA isolated was measured using the Nanodrop One spectrophotometer.

### cDNA synthesis

Reverse transcription of RNA was performed using the MiRCURY LNA RT Kit (Qiagen, 339.340). For FFPE samples, the RNA concentration was adjusted to 5 ng/µl in RNAse-free water and 2 µl was then added to reactions including 0.5 µl cDNA synthesis spike-in control mix, containing UniSp6 according to the manufacturer’s instructions. For EV samples, 2 µl of template RNA was added to each reaction along with 0.5 µl cDNA synthesis spike-in control mix containing UniSp6 according to the manufacturer’s instructions. Samples were incubated for 60 min at 42 °C, 5 min at 95 °C and then cooled to 4 °C. cDNA was stored at -20 °C until the PCR step.

### Quantitative reverse transcription PCR (RT-qPCR)

qRT-PCR reactions were performed using the MiRCURY LNA SYBR Green PCR kit (Qiagen, 339,345), MiRCURY LNA miRNA custom PCR panels in a 96 well format and an ABI7500 Fast Real-Time PCR system. A detailed literature search using PubMed identified a list of miRNAs involved in proliferation, drug resistance, cell growth, metastasis, and invasion in human breast cancer. This list was then cross referenced with the feline miRNAome to identify a list of miRNA candidates with potential relevance in feline mammary cancer. For the initial miRNA screen of *Cohort 1*, the custom PCR panels contained assays for detection of (i) the RNA isolation spike-in controls, (ii) the cDNA synthesis spike-in controls and the (iii) interplate calibrator to identify variations between runs. A list of miRNAs analysed can be found in the Supplementary Material, Table 1. RT-qPCR reactions were prepared according to the manufacturer’s protocol. Similarly, for *Cohort 2*, RT-qPCR of both the FFPE samples and EV samples were carried out using MiRCURY LNA miRNA custom PCR panels designed to target the 8 miRNAs of interest. The plates were in a 96-well format. Briefly, a master mix of 2X MiRCURY SYBR Green Master Mix, ROX and RNase-free water was prepared. The pool was then aliquoted into microcentrifuge tubes where cDNA was added. For EV samples, 0.475 µl of cDNA was added to each well. The mixture was homogenised thoroughly and briefly centrifuged before distributing 10 µl into individual PCR wells. PCR plates were sealed, centrifuged for 1 min at 1000 × g and subjected to real-time PCR amplification according to the protocol, including 2 min heat activation at 95 °C, 40 amplification cycles at 95 °C for 10 s and 56 °C for 60 s. For each run, the Ct value was calculated, and the threshold set manually to fall within the exponential phase of the reaction for all assays. A target miRNA was regarded as undetectable if the recorded Ct value was greater than 35.

### Nanoparticle tracking analysis (NTA)

Nanoparticle tracking analysis (NTA) was carried out using a NanoSight NS300 system (Malvern Technologies, Malvern, UK), configured with a 488 mm later and a high sensitivity CMOS camera. Size distribution and concentration were determined from each feline EV sample following isolation by SEC. Samples were diluted (1:25 dilution) in sterile filtered PBS and loaded into the sample chamber. For each run, samples were analysed using a flow rate of 50 at 25 °C, camera level 12 and screen gain 2. Five × 1 min videos were captured for each sample. Instrument calibration was verified daily using 100 nm polystyrene latex calibration nanoparticles (Malvern Technologies, UK). Data files were analysed using the NTA 4.1 software with a detection threshold of 10 and a bin size of 2. Total EVs per ml and modal size were determined and data was visualised using GraphPad Prism 9.

### EV Protein isolation and quantification

To establish the protein concentration of each EV sample, 50 µl of EVs were collected from each feline patient and lysed using 10 µl RIPA buffer (50 mM Tris HCL pH7.4, 150 mM NaCl, 1% NP40, 1% EDTA, 1 mM phenylmethylsulfonyl, 1% protease inhibitor and 1% phosphatase inhibitor). Samples were left on ice for 30 min, vortexing every 10 min. Samples were sonicated for 3 min and then centrifuged at 10,000 × g for 30 min at 4 °C. Samples were sonicated for 3 min. Protein concentration was carried out using the Pierce BCA Protein Assay Kit (ThermoFisher Scientific: 23,225) in duplicate according to the manufacturer’s instructions. A 25 µl aliquot of EV lysate was added to a 96-well microplate, followed by 200 µl of BCA reagent (ThermoFisher, USA). The plate was subsequently incubated in light exclusion for 30 min at 37 °C and absorbance was measured at 562 nm. The protein concentration was determined from a BSA standard curve and the data was visualised using GraphPad Prism 9.

### SDS-PAGE and western blot

Feline EVs were analysed according to the MISEV 2018 guidelines by western blot analyses^29^. Each sample (n = 2) was concentrated using Amicon Ultrafiltration 10 k filters and lysed using RIPA buffer (as detailed above). Four µg of protein was mixed with 5 µl of 1 × blue loading buffer (NewEngland BioLabs, B7703S) and 1.25 M DTT (reducing agent) and heated to 95 °C for 5 min. Proteins were run on an 8–12% SDS NuPAGE Bis–Tris gel (Invitrogen, NP0326BOX). These gels were run in NuPAGE MOPS SDS running buffer at 200 V for 42 min. Resolved proteins were then transferred to a 0.22 µM nitrocellulose membrane using a BioRad blotting system at a constant 50 V for 75 min. Ponceau stain was carried out to ensure effective transfer (Supplementary Information, Figure 3). The membranes were subsequently blocked for 1 h at room temperature in 1X TBS containing 5% (w/v) bovine serum albumin. Proteins were detected by cutting the membrane according to molecular weights and incubation of individual sections with primary antibodies; CD63 (abcam, ab271286, 1/1000), Calnexin (abcam, ab112995, 1/2000), HSP70 (abcam, ab181606, 1/1000), APOA1 (abcam, ab211472, 1/100) in blocking solution for 48 h at 4 °C. They were then incubated for 4 h at room temperature Due to the lack of availability of feline-specific antibodies, antibodies specific to humans were chosen based on previously published methods^30^. Following 3 X 5-min washes in TBS-T with 0.1% Tween, membranes were incubated in the appropriate dilution of IRDye800-conjugated goat anti-rabbit IgG and IRDye680- conjugated goat anti-mouse IgG secondary antibodies (Li-COR Biosciences) diluted in 5% milk for 1 h at room temperature. The blots were then washed 5 times for 5 min. Proteins were visualised by scanning the membrane with an Odyssey CLx Infrared Imaging System (Li-COR Biosciences) with both 700 and 800 nm channels automatically adjusting the exposure for optimum detection.

### Transmission electron microscopy

EVs from FMA samples and control samples were concentrated from 400 µl. Ten µl of isolated EVs were placed on a formvar carbon-coated copper EM grid for 60 min. Vesicle-coated grids were washed three times with PBS and then fixed using 2.5% glutaraldehyde for 10 min. After washing in distilled water, the grids were stained in 2% uranyl acetate for 15 min and embedded with methyl cellulose-UA for 10 min on ice. Excess cellulose was removed, and samples were air dried for 20 min. Transmission electron microscopy was performed using an FEI Tecnai 120 microscope, operating at an accelerating voltage of 120 kV. Images were taken of the entire field, at 87000X and individual vesicles in that given field were observed at 135000X.

### Statistical analysis

For the initial screen of *Cohort 1*, analysis was carried out using the Gene Globe software (Qiagen). Following distribution analysis using a Shapiro–Wilk test, data were normalised by mean global normalisation of common targets and a paired t-test was carried out. Eight miRNAs were selected using the software for further analysis based on *p* < 0.09 (Supplementary Information, Table 2). Following the initial screen, *Cohort 2* was tested for 8 miRNAs of interest. Normality distribution testing was carried out for all samples using a Shapiro–Wilk test. For FFPE samples, data were normalised using the global mean of common targets. For paired FFPE samples, a non-parametric Wilcoxon rank test was carried out. To analyse independent EV samples, a Mann–Whitney test was carried out (Fig. 3). Analysis was carried out using qBase + software. The mean particle concentration (EV number) was also inputted into the qBase + software. The software then calculated a normalisation factor and EV samples were normalised against the mean particle concentration as previously published^31^. To investigate the strength of the biological differences observed, the effect size (**d**) was calculated using Cohen’s d^32^. Cohen suggested that d = 0.2 is considered a small effect size, 0.5 represents a medium effect size and 0.8 represents a large effect size. Where statistical significance was not reached, a power calculation utilising the effect size was carried out using G-Power (version 3.1). Unless otherwise stated, the graphs represent the mean + /− SEM. Data were analysed and visualised using GraphPad Prism 9.

### Proteomic sample preparation

Concentrated EVs (500 µl) were resuspended in 20 µl PBS. A 40 µl aliquot of 8 M Urea/50 Mm Tris HCL was added to 20 µl of sample. The protein samples were reduced by adding 8 mM DTT, and mixing (thermomixer 1000 rpm, 30 °C) for 60 min and carboxylated by adding 20 mM iodoacetamide and mixing (thermomixer 1000 rpm, 30 °C) for 30 min in light exclusion. The samples were then diluted with 50 mM Tris HCL to bring the urea concentration below 2 M. the Urea concentration must be below 2 M to prevent inhibition of trypsin. Each sample was digested overnight with sequencing-grade trypsin in a 1:20 enzyme-to-substrate ratio (Promega, V5111). Digestion was terminated by adding formic acid to 1% final concentration. Following trypsin digestion, the samples were cleaned using C18 HyperSep SpinTips (Thermo Scientific).

### Mass spectrometry

Following trypsin digestion, the samples were cleaned using C18 HyperSep SpinTips (Thermo Scientific). Samples were run on a Bruker timsTof Pro mass spectrometer connected to an Evosep One liquid chromatography system. Tryptic peptides were resuspended in 0.1% formic acid and each sample was loaded on to an Evosep tip. The Evosep tips were placed in position on the Evosep One, in a 96-tip box. The autosampler is configured to pick up each tip, elute and separate the peptides using a set chromatography method (30 samples a day)^33^. The mass spectrometer was operated in positive ion mode with a capillary voltage of 1300 V, dry gas flow of 3 l/min and a dry temperature of 180 °C. All data was acquired with the instrument operating in a data dependent analysis parallel accumulation serial fragmentation mode (dda-PASEF). Trapped ions were selected for ms/ms using parallel accumulation serial fragmentation (PASEF). A scan range of (100–1700 m/z) was performed at a rate of 5 PASEF MS/MS frames to 1 MS scan with a cycle time of 1.03 s^34^.

Prior to creating a data independent analysis (DIA) method, a pool of the combined trypsin digested samples was analysed using dda-PASEF method described above. The resultant file was used to create the dia-PASEF method within Bruker timsControl software (Supplementary Information, Figure 1). The scan mode “dia-PASEF” was selected and the pooled sample dda-PASEF file was opened in the window editor in the MS/MS tab. Once opened the adjustable rhomboid was used to select the area of the heat map where the identifiable peptides (central region in the heat map containing peptides with charge states from + 2 to + 5) can be found. All data was acquired using data independent analysis parallel accumulation serial fragmentation (dia-PASEF). In summary the dia-PASEF settings used were; mass width 25.0 Da, mass overlap 0.0, mass steps per cycle 35, mobility overlap 0.00, mass range 327.0–1206.4 m/z, mean cycle time estimate 3.44 s^35^.

### Proteomic data analysis

The dda-PASEF raw data was searched against the *Felis catus* subset for feline samples of the Uniprot Swissprot database (reviewed) using the search engine Maxquant (release 2.0.2.0) using specific parameters for trapped ion mobility spectra data-dependent acquisition (TIMS DDA). Each peptide used for protein identification met specific Maxquant parameters. Specifically, only peptide scores that corresponded to a false discovery rate (FDR) of 0.01 were accepted from the Maxquant database search.

Data acquired using dia-PASEF was analysed using DIA-NN 1.8 (Data-Independent Acquisition by Neural Networks)^36^. The *Felis catus* subset from the Uniprot Swissprot database (reviewed) was used to generate a spectral library within DIA-NN (library free mode). Specific search settings included cysteine carbamidomethylation enabled as a fixed modification, N-terminal methionine excision enabled, maximum missed cleavages 1, min precursor + 1, max precursor + 4, Neural network classifier was set to double-pass mode, cross run normalisation was set to retention time dependent and library generation was set to smart profiling. All output was filtered at 0.01 FDR^37^.

To demonstrate the suitability of the DIA database, the identifications from DDA and DIA were compared, and the overlap was examined using Microsoft Excel (Supplementary Information, Figure 6). Using the DIA data, the normalised protein intensity of each identified protein was used for label-free quantitation (LFQ). LFQ values were log2 transformed. For statistical analysis, only proteins identified in 50% of at least one group were included. Differences in protein expression were determined using an unpaired t-test with an FDR of 5% and a minimal fold change of 1 within the Perseus software^38^.

## Results

A detailed literature review using PubMed identified 90 miRNAs of clinical significance in human breast cancer pathology (Supplementary Information, Table 2). Initially, following tumour resection excess FFPE tissue was collected from 10 feline patients with a diagnosis of FMA to form *Cohort 1.* Eight miRNAs with significantly altered expression in tumour tissue compared to margins were selected for further studies in FMA patients. *Cohort 2* consisted of FFPE tissue samples and plasma collected from 10 patients with FMA. Ten control plasma samples were also collected from healthy felines. The clinical characteristics of these patients are included in Table 1.

**Table 1:** Demographic information of feline patients with FMA recruited to the study.

Demographic details	Cohort 1 FFPE	Cohort 2 FFPE & Plasma	Healthy controls plasma
Age			
(years, median, (min–max))	10 (6–14)	10 (6–17)	1 (0.5–4)
Sex n (%)			
Female	10 (100%)	10 (100%	10 (100%)
Male	–	–	–
Neuter status n (%)			
Entire	7 (70%)	–	1 (10%)
Neutered	3 (30%)	10 (100%)	9 (90%)
Breed n (%)			
DSH	8 (80%)	2 (20%)	–
DLH	1 (10%)	–	–
Ragdoll	1 (10%)	–	–
Unknown	–	8 (80%)	10 (100%)
Grade n (%)			
I	4 (40%)	3 (30%)	–
II	2 (20%)	4 (40%)	–
III	4 (40%)	3 (30%)	–
Lymphatic invasion n (%)			
Yes	2 (20%)	5 (50%)	–
No	8 (80%)	5 (50%)	–

DSH: Domestic Short Haired; DLH: Domestic Long Haired.

### RT-qPCR highlighted 8 miRNAs of interest for further investigation

The expression of 90 miRNAs were examined by RT-qPCR in matched tumour tissue (n = 10) and margins (n = 10) from each patient with FMA in* Cohort 1*. Histological sections of a representative tumour and respective margin are available in Supplementary Information, Figure 7. While all 90 miRNAs were interesting (Supplementary Information, Figure 2), a paired t-test demonstrated specific differences in the expression of 8 miRNAs in tumour tissue compared to tumour margins. To acknowledge our small sample size, miRNAs with a *p*-value < 0.09 were selected for further investigation and are shown in Table 2.

**Table 2:** Differentially expressed miRNAs in matched tumour tissue compared to margins in 10 felines with FMA in Cohort 1. These miRNAs were selected for further investigation.

miRNA ID	Sequence	*P*-Value
fca-miR-15b-5p	UAGCAGCACAUCAUGGUUUACA	0.034
fca-miR-20a-5p	UAAAGUGCUUAUAGUGCAGGUA	0.062
fca-miR-96-5p	UUUGGCACUAGCACAUUUUUGCU	0.062
fca-miR-378-5p	CUCCUGACUCCAGGUCCUGUGU	0.067
fca-miR-365a-3p	UAAUGCCCCUAAAAAUCCUUAU	0.071
fca-miR-22-3p	AAGCUGCCAGUUGAAGAACUGU	0.078
fca-miR-200c-3p	UAAUACUGCCGGGUAAUGAUGGA	0.082
fca-miR-31-5p	AGGCAAGAUGCUGGCAUAGCUGU	0.082

### EV isolation and characterisation

**Figure 2:**
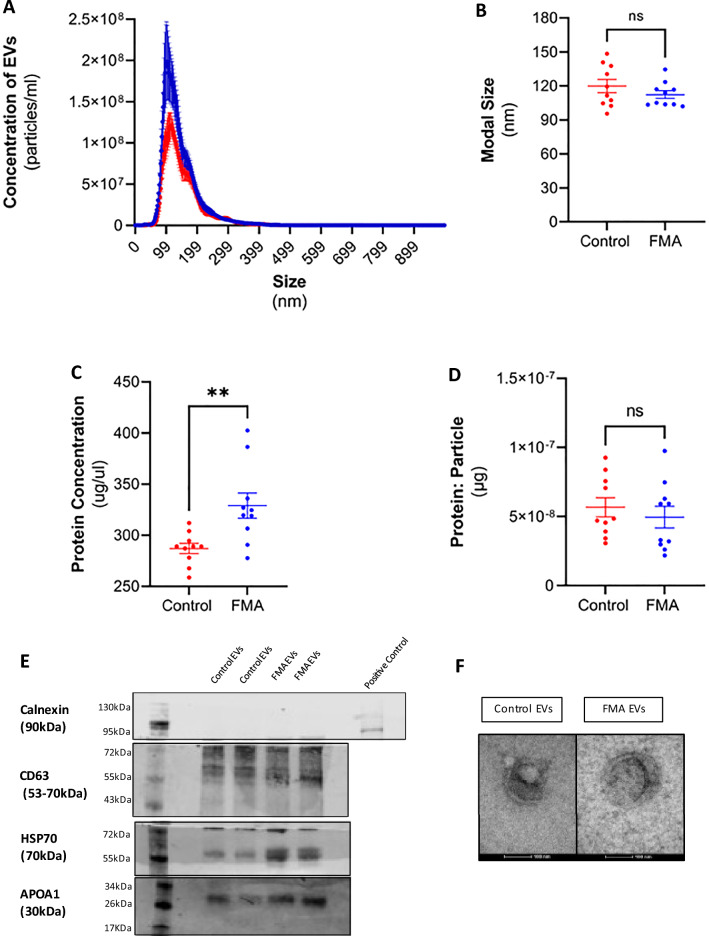
Isolation and characterisation of EVs isolated from the plasma of feline patients with FMA and healthy control felines. EVs were isolated from plasma by SEC. EVs were quantified and their size distribution was determined by nanoparticle tracking analysis (NTA) and the protein content of isolated EVs was determined using a Pierce BCA protein assay. (**A**) NTA demonstrates an increased concentration of EVs isolated from the plasma of patients with FMA (blue) compared to healthy control plasma (red) (*Independent t-test, P* < *0.0001*). (**B**) No significant difference was observed in the modal size of EVs isolated from patients with FMA compared to healthy controls (*Independent t-test, p* = *0.1168*). (**C**) The total protein concentration is significantly increased in EVs from patients with FMA compared to healthy controls (*Independent t-test, p* = *0.003*). (**D**) The protein content of EVs was normalised to particle number. The total protein per particle was similar in EVs from FMA compared to control EVs (*Independent t-test, p* = *0.505*). (**E**) EVs were subjected to western blot analysis for recognised EV markers according to the MISEV guidelines 2018^29^. Due to limitations in sample volume and difficulties with antibody specificity previously demonstrated^30^, EVs from FMA and controls were subjected to western blot analysis for Calnexin, CD63, HSP70 and APOA1 (n = 2). EVs from the plasma of patients with FMA and healthy controls were negative for Calnexin, showed similar levels of CD63, and HSP70 and had similar levels of lipoprotein contamination as demonstrated by APOA1. (**F**) Shows the structure of a single EV from healthy controls and felines with FMA by TEM (magnification: 135,000X, Scale bar: 100 nm).

Plasma samples were collected from 10 patients with FMA and 10 controls in *Cohort 2 (*Table 1*)*. EVs were isolated from patient plasma by size exclusion chromatography (SEC) and characterised using previously published protocols^30^. In Fig. 2A, nanoparticle tracking analysis (NTA) demonstrated that plasma from feline patients with FMA contains significantly more EVs than healthy controls. However, there was no change in the modal size (Fig. 2B) of EVs between groups. The protein concentration of isolated EVs from patients with FMA was also increased compared to controls (Fig. 2C). When the protein content per EV particle was calculated, there was no significant difference observed, demonstrating that the quantity of protein contained inside EVs was similar between groups. Western blot analyses of 4 μg of EV proteins from patients with FMA (n = 2) and controls (n = 2) was carried out according to provide proof of principle according to the MISEV 2018 guidelines^29^. These plasma derived EVs were negative for endoplasmic reticulum protein Calnexin and the presence of CD63 demonstrates the well-known lipid bilayer structure of EVs. Feline EVs were also positive for the cytosolic protein HSP70. Co-isolation of lipoproteins is a common caveat of EV isolation. Therefore, the presence of APOA1 demonstrates that a limited number of lipoproteins are being co-isolated with EVs (Fig. 2D). *The uncropped blot is available in the Supplementary Material, Figure *3. Figure 2E demonstrates cup-shaped vesicles, with morphology and size compatible with EVs using TEM.

### miR-20a and miR-15b can differentiate between tumours and margins as well as identify felines with FMA based on their plasma EVs

**Figure 3:**
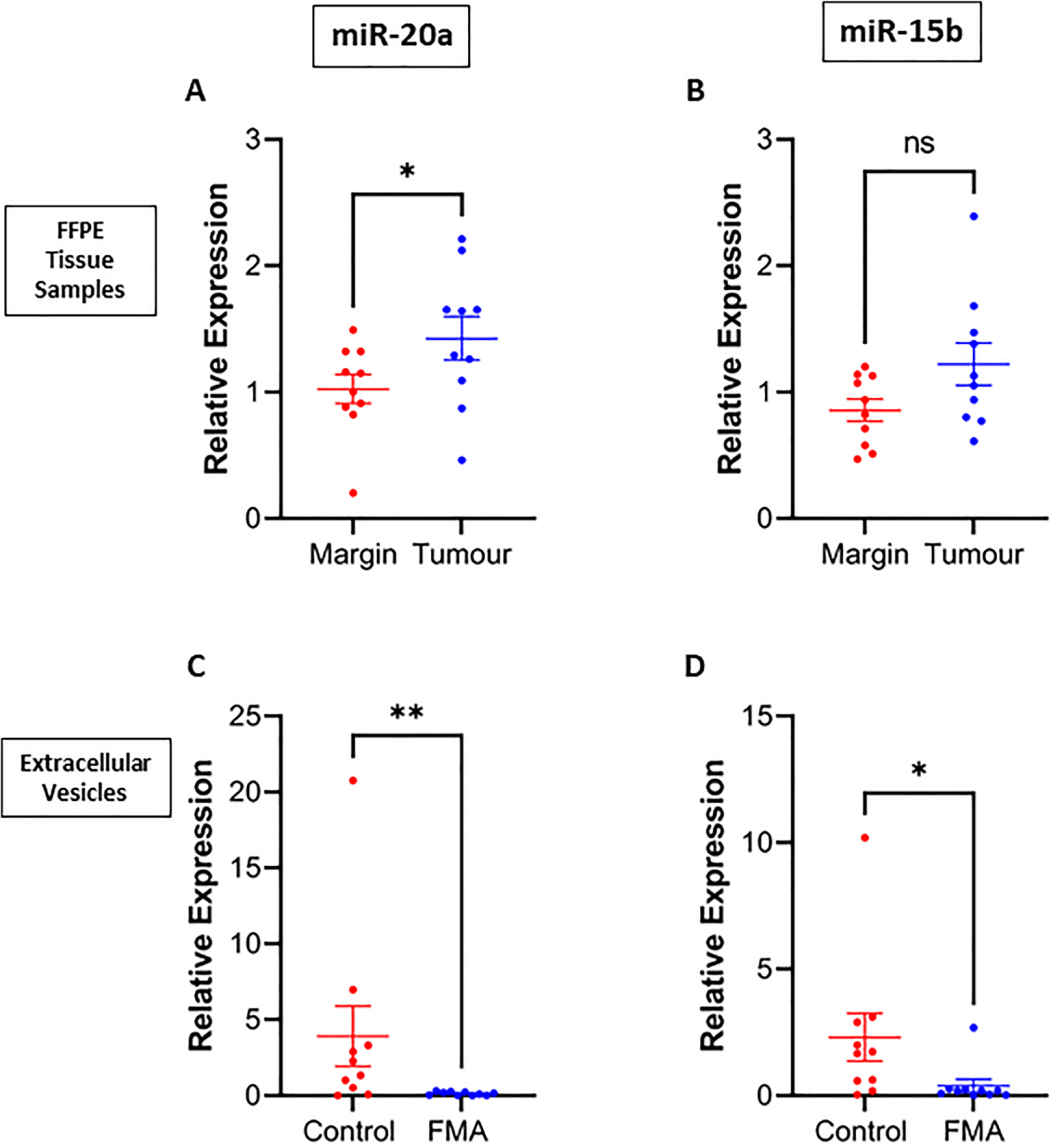
Increased expression of miR-15b and miR-20a in FMA tumour tissue is reflected by significantly decreased expression in plasma EVs from patients with FMA compared to controls. Matched tumour tissue (n = 10) and tumour margins (n = 10) isolated from felines with FMA were subjected to RT-qPCR for 8 miRNAs of interest. The effect size (d) demonstrates that the magnitude of these observed differences was large and potentially holds practical significance. (**A**) Demonstrates significantly increased expression of miR-20a in tumour tissue compared to tumour margins (*Wilcoxon test, p* = *0.014, d* = *0.571)*), while (**B**) shows no significant difference in expression of miR-15b in tumour tissue compared to tumour margins (*Wilcoxon test, p* = *0.193, d* = *0.8441*). From each feline with FMA (n = 10), a plasma sample was also collected. Control plasma samples were collected from healthy felines for comparison (n = 10). Isolated EVs were subjected to RT-qPCR for 8 miRNAs of interest. Data were normalised by EV number^31^. Due to the limited amount of RNA inside EVs, only 2 miRNAs were detected in all EV samples (n = 20). (**C**) demonstrates significantly decreased miR-20a expression in EVs from patients with FMA compared to controls (*Mann–Whitney, p* = *0.0041, d* = *0.852)*, while (**D**) also demonstrates significantly decreased miR-15b expression in EVs from FMA patients compared to controls (*Mann Whitney, p* = *0.012, d* = *0.8773*). The effect size (d) demonstrates that the magnitude of these observed differences was large and potentially holds practical significance in early diagnosis of FMAs.

In* Cohort 2*, the expression of 8 miRNAs previously selected in *Cohort 1* was investigated in FFPE and plasma EVs from 10 felines with FMA. miR-20a and miR-15b were detected in all EV and FFPE samples. Within the FMA group, expression levels of miR-20a and miR-15b in FFPE tumour, margins and EVs were also compared between tumour grades and lymphatic invasion status (*Supplementary Information,* *Figures* 4*and* 5). RT-qPCR of matched tumour tissue and margins (Fig. 3) demonstrated that miR-20a was significantly increased in tumour tissue compared to margins. Figure 3B shows that miR-15b was also increased in tumour tissue compared to margins, although not significant (*p* = 0.06). Power analysis demonstrated that increasing the sample size to 14 felines would have yielded a statistically significant increase in miR-15b expression in tumour tissue compared to margins^32^. Interestingly, expression levels of both miR-20a (Fig. 3C) and miR-15b (Fig. 3D) were significantly decreased in the plasma EVs from patients with FMA compared to healthy controls. The strength of the observed differences was complemented by the effect size (d)^32^. Interestingly, Cohen’s d shows that the observed differences in miRNA expression in FFPE for miR-20a were medium while miR-15b had a large effect size. In EVs, the large effect size shows that miR-20a and miR-15b indicates a strong relationship of potentially practical significance between miRNA expression and FMA.

### The proteomic profile of plasma derived EVs can identify felines with FMA and may also reflect the miRNA contents of EVs

EVs from the plasma of felines with FMA and controls were digested and analysed by LC–MS/MS. As there have been limited studies examining the feline proteome, peptides were quantified using data-independent acquisition (DIA). To confirm the reproducibility of DIA identifications, samples were cross-examined using data-dependent acquisition (DDA). Upon comparison, 90% of proteins identified by DDA were also detected by DIA (*Supplementary Material, Figure* 6). Therefore, DIA data were analysed for biological significance. LC–MS/MS data were analysed using Perseus (version 1.6.2.1)^38^. Figure 2A. demonstrates that the proteomic content can distinguish EVs from patients with FMA and EVs from healthy felines, while Fig. 2B. highlights the significantly different levels of proteins in EVs from felines with FMA compared to controls. Due to the lack of feline-specific databases, miRTarBase was used to identify the protein targets of miR-20a and miR-15b^39^. Six significantly decreased proteins in EVs from FMA are known targets of miR-20a and miR-15b. Within the FMA group, expression levels of protein targets were also compared between tumour grades and lymphatic invasion status (*Supplementary Information, Table *3).

## Discussion

Feline mammary adenocarcinoma (FMA) is aggressive and metastatic with poor survival for patients diagnosed. Comparative medicine involves relating biological similarities and differences among species to examine naturally occurring diseases in humans and animals to better understand the disease. Comparative medicine is an upcoming area of interest for many researchers worldwide as it also allows basic scientific research to be translated more rapidly to the clinical setting. The similarities and differences in humans and felines with FMAs have been discussed in detail in the literature^1^. However, research carried out on human breast cancer is quite rarely translated to naturally occurring cancer in other animals. The felines recruited to the study represent the true population of felines with mammary cancer presenting to the veterinary practices. Studies have shown that 75% of FMAs display the “triple-negative” or “basal-like” subtype regardless of neuter status^40^. These FMA tumours are ER negative, PR negative and do not overexpress HER2, indicating that their development is not affected by female hormone levels. Therefore, this statistic is also expected apply to felines recruited, suggesting that the majority are not affected by female hormone levels.

Using a comparative medicine approach, we selected 90 miRNAs that held biological significance such as involvement in metastasis, cell growth or proliferation in human breast cancer. These miRNAs were then cross-referenced against the feline miRNA database and the feline-specific assays were developed. The expression of these 90 miRNAs was analysed in 10 feline tissue samples from *Cohort 1* (Table 1). Expression was compared between tumour tissue and tumour margins. Although there is only a single study published detailing the miRNA profile of feline tissues, mammary tissue was not included in that study^27^. Comparing the expression of 90 miRNAs gave interesting results and highlighted 8 miRNAs of particular significance (Table 2). miRNAs with *p*-value < 0.09 were chosen, as these miRNAs were considered significant or ‘trending’ towards significance in our initial pilot study. Although *p* = 0.05 is commonly accepted as the threshold for statistical significance, a less stringent threshold for investigation was chosen to recognise the small sample size. Given the exploratory nature of the study, it is important to cast a wide net initially in order to identify promising candidates for further investigation. This study also emphasises the benefit of analysing FFPE and its potential in biological science research. Extracellular vesicles (EVs) are found in the bloodstream of felines during normal and pathological conditions where they carry cargo such as lipids, proteins, and RNA. Conflicting evidence has emerged as to whether EVs found in circulating plasma reflect their parent cell and whether they can accurately predict the disease state^22,41^. Studies have highlighted EVs as a potential tool to aid with the diagnosis of disease, identify therapeutic targets, and predict disease prognosis. Additionally, circulating EVs are easily accessible from plasma or urine and as such offer less invasive mechanisms of disease monitoring in the clinical setting. Despite extensive research into the application of EV research as biomarkers of disease there have been limited studies on EVs in felines in veterinary clinics. Our previous work has established and confirmed that EVs may be isolated from feline plasma and that as with human EVs, they may be characterised according to the MISEV guidelines^29,30^.

The main objective this study was to analyse EVs in feline patients with FMA and to investigate whether EVs may be potential diagnostic and or prognostic markers for this disease. *Cohort 2* consisted of 10 feline patients recruited from primary care veterinary practices. The differences in neuter status between cohort 1 and cohort 2 are acknowledged, however the miRNA profile of felines between cohorts 1 and 2 are not compared. Therefore, it is not expected that feline hormone levels will influence the presented results. Plasma samples and FFPE tissue samples were collected from each patient. Plasma was also collected from healthy control felines. EVs were isolated from plasma by SEC, quantified by nanoparticle tracking analysis (NTA), and their total protein concentration was measured by BCA. Additionally, EVs were subjected to western blot for known EV markers according to our previously published methods^30^. Finally, single particle analysis was performed by TEM. Figure 2A. shows that there were significantly increased EVs present in the plasma from felines with FMA compared to healthy felines, with the majority of EVs isolated in the small EV size range (< 200 nm). Figure 2B. demonstrates that there is no significant difference in the modal size of EVs regardless of whether felines had FMA compared to controls. Total protein concentration demonstrated (Fig. 2C.) that samples from FMA had significantly higher protein concentration than controls. However, when the total protein concentration was normalised to particle number, Fig. 2D. shows that there was no significant difference in the protein concentration per EV particle. This suggests that the total protein concentration per EV particle does not charge regardless of whether the feline has been diagnosed with mammary cancer. Although NTA profiles indicate a significant increase in patients with FMA compared to controls, the use of EV quantification as a biomarker requires further research. Firstly, EVs can be increased in the cancer setting^31^, although evidence is conflicting^42^, and EVs in circulation come from a wide variety of origins, not just cancer, making them a heterogenous population of vesicles^43^. It is important to note that increased concentration of EVs may be due to a variety of reasons such as systemic inflammation, haematological diseases, or the presence of viruses, to name a few. Barlin et al., (2023) reported that only a small proportion of proteins in plasma EVs are from the tumour, highlighting the complexity of EV secretion from the body and the need for complex subpopulation analysis for biomarker discovery. Secondly, there are limitations to current EV quantification methods, such as the minimum detectable sizes of equipment and the inability to distinguish EVs from other types of nanoparticles or lipoproteins. For instance, NTA instrumentation is widely accepted as a method of quantification/ size analysis but cannot distinguish an EV from another type of nanoparticle or lipoprotein. More sophisticated analysis methods such as AF4 could investigate this further^44^, however, they are not widely available for reliable detection of EV subpopulations. Thus, further research is needed to determine the threshold for the increase in EVs and to distinguish cancer EVs from other pathologies before using EV quantification as a biomarker.

Western blot was carried out for known EV markers as per our previously published study^30^. EVs from felines with FMA and controls were negative for calnexin and positive for CD63, HSP70 and had limited APOA1 (Fig. 2E). HSP70 is indicated by the protein band at approximately 72 kDa. However, it is important to note that HSP70 is a member of the HSP subfamily with proteins approximately 70 kDa weight that serve as molecular chaperones in cells. In humans, there are at least 14 different HSP70 proteins that differ in various aspects, such as expression level, subcellular location and inducibility to stress. Not all these isoforms are exactly 70 kDa in humans, and none of them have been studied in felines. Therefore, we hypothesise that the additional bands seen at approximately 60 kDa could be an isoform of HSP70, as seen in human studies^45^. Further research is needed to truly identify these bands as isoforms in feline studies. One of the main limitations when carrying out western blot of EVs from feline plasma is the volume of sample available. For this reason, EVs samples were analysed in duplicate (n = 2). Finally, single EV analysis using TEM shows a single particle in the small EV subtype with a cup-shaped morphology associated with EVs (Fig. 2F).

To investigate the validity of miRNA as biomarkers of FMA, the 8 miRNAs of interest from *Cohort 1* were investigated further in *Cohort 2*. RNA was isolated from matched tumour tissue and margins from 10 patients in *Cohort 2*. *Histological images of the tumours and respective margins have been included in Supplementary Information, Figure *7. The expression level of 8 miRNAs of specific interest with *p* < 0.09 were analysed in both tumour tissue and margins. These miRNAs were selected as they were considered significant or ‘trending’ towards significance in Cohort 1. Additionally, the miRNA expression was also measured in EVs from the plasma of felines with FMA and healthy controls. One of the main limitations of this study is the use of tumour margins as controls to compare to the FMA tumours. In human breast cancer studies, healthy control tissue is generally readily available through breast reduction surgeries^46^. However, this is not the case for feline patients where these mammary surgeries are not carried out in veterinary clinics in the absence of any pathology. Therefore, the only available control tissues for feline studies are tumour margins. Figure 3 shows that miR-20a expression was significantly increased in tumour tissue compared to tumour margins (Fig. 3A). This supports results previously published by Gao et al., demonstrating the involvement of the PTENP1/MiR-20a/PTEN axis in malignant behaviours of human breast cancer cells^47^. MiR-15b expression was also increased in tumours compared to margins, with power analysis demonstrating that increasing the size *of Cohort 2* to fourteen felines would have yielded significantly increased expression of miR-15b expression in tumour tissue compared to controls (Fig. 3B). Previously published studies in humans have demonstrated that miR-15b suppresses PEBP4 expression and in turn contributes to metastasis and chemoresistance of lung carcinoma cells^48^. Further comparison of FMA samples were also conducted based on tumour grade and lymphatic invasion. There was no significant difference in expression levels of miR-20a and miR-15b in FFPE tumour samples regardless of grade or lymphatic invasion. For the first time, these data have highlighted miR-20a and miR-15b as potential mediators of FMA metastasis.

To investigate whether a liquid biopsy may be used to identify these miRNAs mediating metastasis, plasma samples were collected and EVs were isolated from healthy felines and felines with FMA. For the first time, the relative expression of miR-20a and miR-15b in feline plasma derived EVs was explored. The main factor that has limited researchers when studying miRNA cargoes of EVs to date has been the limited sample availability. EVs typically contain very small amounts of miRNA, therefore isolation and subsequent RT-qPCR is challenging. This study has demonstrated successful isolation and RT-qPCR expression analysis of plasma derived EVs. Interestingly, RT-qPCR analysis of plasma derived EVs displays a differential miRNA profile of EVs from patients with FMA compared to healthy controls. Results show that expression of both miR-20a and miR-15b was significantly decreased in EVs from patients with FMA compared to controls. To investigate the size of the observed differences, the effect size was calculated. Cohen’s d^32^ confirmed that the differences in miR-20a and miR-15b expression in EVs have a large effect size (d > 0.8). The large effect size suggests that the significant differences in miRNA expression observed have biological significance.

Based on these interesting results, we have generated hypotheses to be investigated further. The first hypothesis is that miR-20a and miR-15b are involved in FMA tumour progression, therefore less of these miRNAs are being trafficked out of the tumour via the EVs and into the circulation from the tumour of origin. Additionally, the selective packaging of EV cargo is an important concept to consider, as EVs are often described as messenger vesicles that play a role in communication between cells of origin and destination cells. Therefore, the EVs may be functioning as transport vesicles to move the miRNAs and proteins detected from their cell of origin to a recipient cell where they can carry out a function. As such, the usual patterns of increased miRNA corresponding to decreased protein targets may not apply. Alternatively, EVs in the plasma may in fact reflect the miRNA profile of the tumour margins which constitute the tumour microenvironment instead of the miRNA profile of the actual tumour tissue. Stromal cells can make up approximately 90% of a solid tumour, therefore it is possible that in FMA, stromal cells constitute a large proportion of the tumour microenvironment. The tumour stroma mainly consists of the basement membrane, fibroblasts, extracellular matrix, immune cells and vasculature also contributing to the circulating EV populations. In the literature, upregulated miR-15b has been shown to promote breast cancer cell proliferation, migration and invasion^49^, however, a neuroblastoma study by Pathania et al., (2022) demonstrated the role of miR-15b as a tumour suppressor. Patients with decreased miR-15b demonstrated increased stromal cells numbers and significantly decreased genes relating to T-cells and natural killer cells. This suggests that decreased circulating miR-15b inside EVs may be indicative of increased stromal cell populations in the tumour microenvironment and therefore be supporting the growth of tumours. Numerous studies have researched the effect of miR-20a on cancer. Zhang et al., evaluated EV RNAs from multiple myeloma patients who were being treated by immunotherapy^50^. They demonstrated that multiple myeloma patients in the drug resistant group demonstrated downregulation of miR-20a in their EVs, showing that miR-20a may serve as a biomarker for drug resistance in multiple myeloma patients. Although studies are limited, this suggests that decreased EV miR-20a may in fact be an indicator of drug resistance in FMA. Lastly, as EVs have been shown to play a role in regulating immune responses^51^, the reduced genetic material in cancer derived EVs may allow the EVs to avoid recognition by the immune system, thus giving them the ability to promote cancer progression. The mechanism underlying the differences in miR-20a and miR-15b cargoes contained in EVs from felines with FMA compared to controls clearly warrant further investigation. However, we have demonstrated the potential role of miR-20a and miR-15b as circulating biomarkers of FMA.

**Figure 4:**
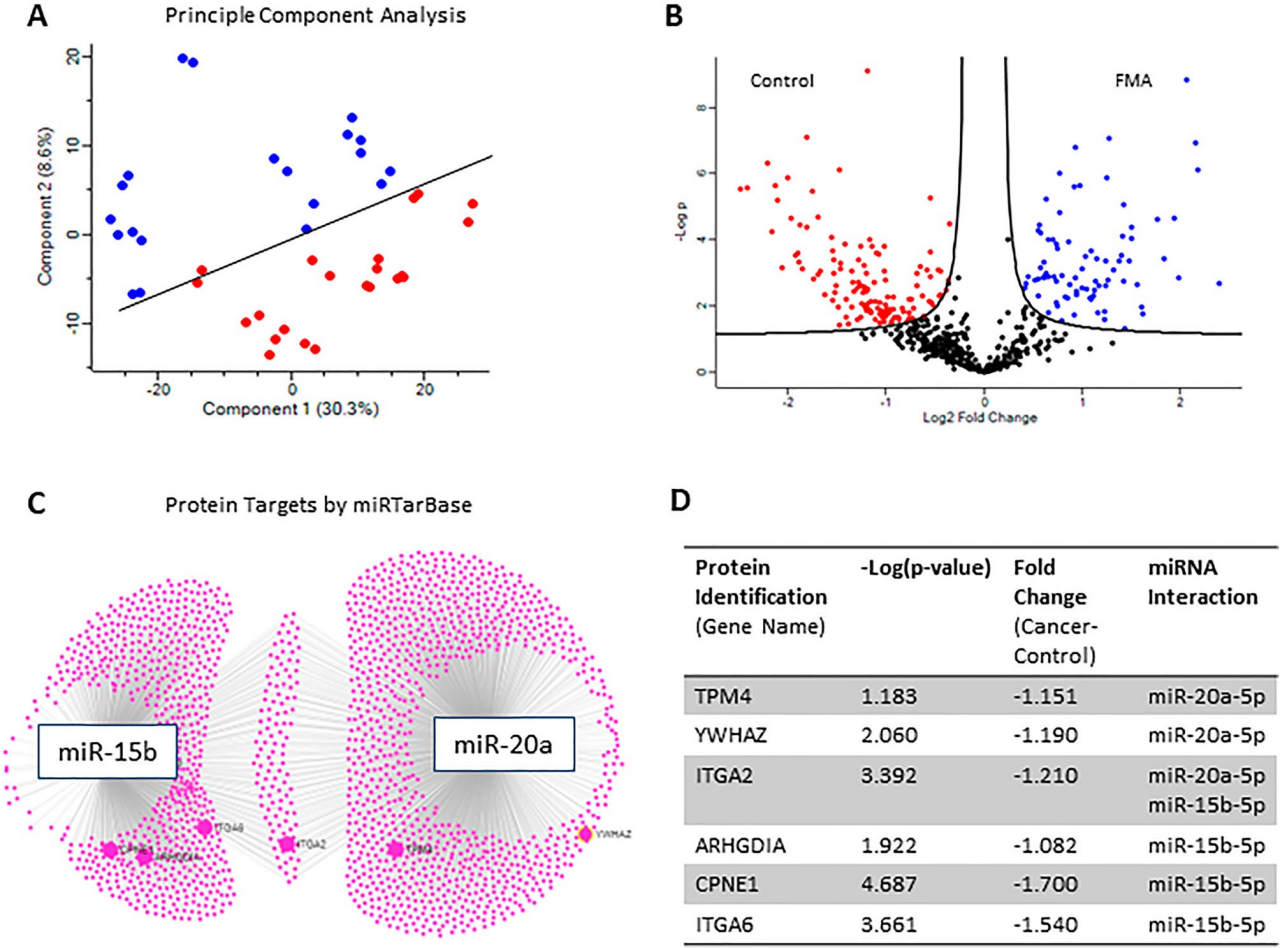
Mass Spectrometry analysis of EVs isolated from the plasma of patients with FMA compared to controls. EVs were isolated from plasma samples taken from felines with FMA and healthy feline controls. Following digestion, EVs were subjected to LC–MS/MS^30^. (**A**) Principle component analysis (PCA) demonstrates that EVs from patients with FMA (blue) can be distinguished from control EVs based on their proteomic profile (red). (**B**) Volcano plot demonstrating the differential encapsulation of proteins in plasma EVs between FMA and controls. Proteins significantly increased in EVs from FMA (55 proteins) were indicated in blue while proteins decreased in FMA (84 proteins) were indicated in red. All other proteins were plotted in black. The x-axis indicates fold-change (log 2 scale) and the y-axis indicates –log significance (*p* < 0.05). miRNet 2.0 and miRTarBase were used to investigate the proteins targeted by miR-15b-5p and miR-20a-5p. miR-15b-5p was shown to target 761 proteins while miR-20a-5p targets 1073 proteins. The protein targets were compared to the significantly altered proteins in EVs from patients with FMA. (**C**) highlights 4 significantly altered EV proteins that are known to be targeted by miR-15b-5p and 3 significantly altered EV proteins targeted by miR-20a-5p. (**D**) Table demonstrating that miR-15b and miR-20a target specific proteins that are significantly decreased in EVs from patients with FMA compared to controls.

Finally, the proteomic profile of EVs from felines with a diagnosis of FMA was compared to control EVs (Fig. 4). Following LC–MS/MS, peptides were quantified using DIA. DIA was used over the classic DDA as it offers potentially deeper coverage of the data. Reliance on the library for identifications means that DIA does not suffer from the stochastic identifications that DDA suffers from. Additionally, the power of this method when applied to oncology research has been highlighted in recent reviews^52^. To confirm the integrity of the proteomic dataset generated, the DIA identifications were compared to DDA identifications (*Supplementary Material, Figure *6) to show that 90% of proteins identified by DDA were also identified by DIA. Based on this, DIA data were analysed for biological significance. In Fig. 4A, principal component analysis demonstrates that the proteome of EVs can be separated into two clear clusters, EVs from patients with FMA and controls. This suggests that felines with FMA may be distinguished from healthy felines based on the proteomic profile of EVs. In clinical practice, this concept may identify patients with FMA using a non-invasive liquid biopsy approach. The differences in the protein content of EVs were then tested at the 5% level of significance. Even though there was no difference between the total protein concentration of EVs in patients with FMA compared to control (Fig. 2D), there were significant differences in the expression of 139 individual proteins. Figure 2B. shows the proteins that were significantly increased in EVs from FMA in blue and decreased in red. To investigate the significance of these proteins and to highlight potential biomarkers of FMA, the protein targets of miR-20a and miR-25b were investigated. According to miRTarBase^39^ miR-20a targets 1073 proteins, while miR-15b targets 761 proteins (Fig. 4C). When these proteins were compared to the significantly altered proteins in EVs from FMA compared to controls, 6 proteins known to be targeted by miR-20a and miR-15b were significantly decreased in EVs from felines with FMA compared to controls (Fig. 4D). Interestingly, ITGA2 is targeted by both miR-20a and miR-15b. ITGA2 silencing has previously been shown to stimulate breast cancer migration^53^. As expected, the decrease in miR-20a and miR-15b expression in EVs from patients with FMA is confirmed by the significant decrease in the protein targets in EVs from FMA. Specifically, ITGA2 is a known target of miR-20a and miR-15b, therefore representing an interesting panel of molecular targets warranting further investigation in felines with mammary adenocarcinoma.

This study investigated the miRNA expression of feline mammary adenocarcinoma tissue and demonstrated that FFPE samples hold huge relevance for basic biological research in the veterinary field. Additionally, we have shown that miRNAs can distinguish tumour tissue from margins. One of the main limitations of research in veterinary medicine and FMA specifically is the low accrual rate to research studies. The veterinary field would benefit hugely from developing biobanks containing tissue, plasma and urine samples collected in an ethical manner to be used in veterinary research. As EVs gain traction as biomarkers of disease worldwide, we have shown the EVs isolated from felines with FMA also hold potential as biomarkers in veterinary medicine. In the EV field, the normalisation of RT-qPCR has posed great challenges for researchers. As there have been no published studies to date on the miRNA profile of EVs from feline patients, relative normalisation was not possible. Based on the normalisation method used by Moloney et al., we were able to normalise miRNA expression to the EV number^31^ and thus generate meaningful results. This highlights the need to study EVs in patients with naturally occurring diseases in veterinary medicine.

## Conclusion

In conclusion, this study has demonstrated for the first time that miRNAs are detectable in feline mammary tissue. Specifically, we have shown that miRNA expression differs between mammary tumour tissue and tumour margins. To identify a potential non-invasive biomarker, EVs have been isolated and characterised from the plasma of felines with FMA. This study demonstrated that circulating EVs reflect the miRNA profile of mammary tumours, with a significant decrease in miR-15b and miR20a in circulating plasma EVs shown to be associated with FMA. Additionally, the proteomic content of EVs can distinguish felines with FMA from healthy control felines. The protein targets of miR-15b and miR-20a also significantly decreased in plasma EVs from patients with FMA compared to controls. In summary, this study provides the veterinary community with a novel panel of markers reflecting FMA tumours that are easily detectable in circulating plasma EVs and may have the potential to inform future diagnostic tests for FMA in a non-invasive manner in the clinic.

## Supplementary Information

Supplementary Information.

## Data Availability

The datasets generated and analysed are available from the corresponding author upon request.
