# Supplementary material for: The protein and miRNA profile of plasma extracellular vesicles (EVs) can distinguish feline mammary adenocarcinoma patients from healthy feline controls
Source: Sci Rep. 2023 Jun 6;13:9178. doi: 10.1038/s41598-023-36110-7 (PMC10244388; doi:10.1038/s41598-023-36110-7)

**Supplementary Material:**


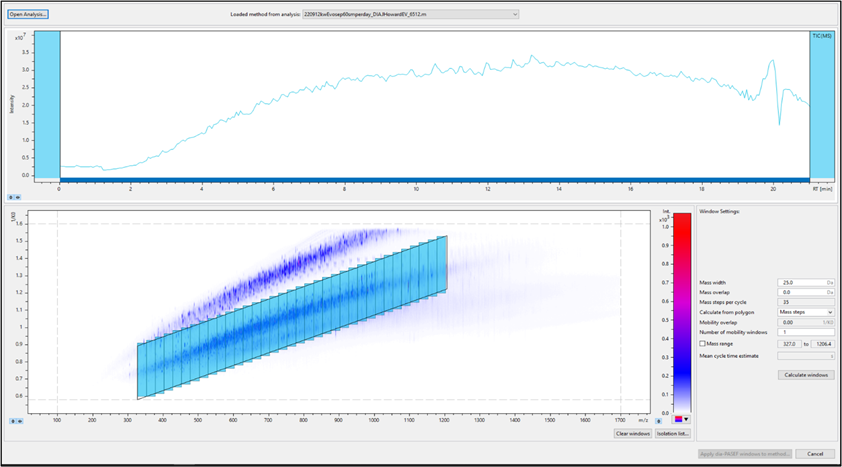


**Figure S1. Bruker timsControl software (Window editor) was used to create the DIA-PASEF method.**

**Table S1: Specific miRNA targets (n=90) investigated in Cohort 1 were selected according to PubMed search based on their association with human breast cancer. Details of the previous validation status of the target miRNA are also included.**

| **Target miRNA** | **Sequence** | **Qiagen GG Cat No.** | **Previous**  **Validation status** |
| --- | --- | --- | --- |
| fca-let-7a-5p | UGAGGUAGUAGGUUGUAUAGUU | YP00205727 | Wet-lab validated |
| fca-let-7c-5p | UGAGGUAGUAGGUUGUAUGGUU | YP00204767 | Wet-lab validated |
| fca-let-7e-5p | UGAGGUAGGAGGUUGUAUAGUU | YP00205711 | Wet-lab validated |
| fca-let-7f-5p | UGAGGUAGUAGAUUGUAUAGUU | YP00204359 | Wet-lab validated |
| fca-let-7g-5p | UGAGGUAGUAGUUUGUACAGUU | YP00204565 | Wet-lab validated |
| fca-let-7i-5p | UGAGGUAGUAGUUUGUGCUGUU | YP00204394 | Wet-lab validated |
| fca-miR-100-5p | AACCCGUAGAUCCGAACUUGUG | YP00205689 | Wet-lab validated |
| fca-miR-10a-5p | UACCCUGUAGAUCCGAAUUUGU | YP02102486 | In silico validated |
| fca-miR-10b-5p | UACCCUGUAGAACCGAAUUUGU | YP02104576 | In silico validated |
| fca-miR-125b-1-3p | ACGGGUUAGGCUCUUGGGAGCU | YP00204400 | Wet-lab validated |
| fca-miR-125b-5p | UCCCUGAGACCCUAACUUGUG | YP02114394 | In silico validated |
| fca-miR-128-3p | UCACAGUGAACCGGUCUCUUU | YP00205995 | Wet-lab validated |
| fca-miR-129-5p | CUUUUUGCGGUCUGGGCUUGC | YP00204534 | Wet-lab validated |
| fca-miR-130a-3p | CAGUGCAAUGUUAAAAGGGCAU | YP00204658 | Wet-lab validated |
| fca-miR-130b-3p | CAGUGCAAUGAUGAAAGGGCAU | YP00204317 | Wet-lab validated |
| fca-miR-132-3p | UAACAGUCUACAGCCAUGGUCG | YP00206035 | Wet-lab validated |
| fca-miR-1-3p | UGGAAUGUAAAGAAGUAUGUAU | YP00204344 | Wet-lab validated |
| fca-miR-140-5p | CAGUGGUUUUACCCUAUGGUAG | YP00204540 | Wet-lab validated |
| fca-miR-148a-3p | UCAGUGCACUACAGAACUUUGU | YP00205867 | Wet-lab validated |
| fca-miR-152-3p | UCAGUGCAUGACAGAACUUGGG | YP02105375 | In silico validated |
| fca-miR-155-5p | UUAAUGCUAAUCGUGAUAGGGGU | YP00204308 | Wet-lab validated |
| fca-miR-15a-5p | UAGCAGCACAUAAUGGUUUGUG | YP00204066 | Wet-lab validated |
| fca-miR-15b-5p | UAGCAGCACAUCAUGGUUUACA | YP00204243 | Wet-lab validated |
| fca-miR-16-5p | UAGCAGCACGUAAAUAUUGGCG | YP00205702 | Wet-lab validated |
| fca-miR-17-5p | CAAAGUGCUUACAGUGCAGGUA | YP02101418 | In silico validated |
| fca-miR-181a-5p | AACAUUCAACGCUGUCGGUGAGU | YP00206081 | Wet-lab validated |
| fca-miR-181b-5p | AACAUUCAUUGCUGUCGGUGGGU | YP00204530 | Wet-lab validated |
| fca-miR-181c-5p | AACAUUCAACCUGUCGGUGAGU | YP00204683 | Wet-lab validated |
| fca-miR-181d-5p | AACAUUCAUUGUUGUCGGUGGGU | YP00204789 | Wet-lab validated |
| fca-miR-182 | UUUGGCAAUGGUAGAACUCACACUG | YP02106325 | In silico validated |
| fca-miR-186-5p | CAAAGAAUUCUCCUUUUGGGCU | YP00206053 | Wet-lab validated |
| fca-miR-18a-5p | UAAGGUGCAUCUAGUGCAGAUAG | YP00204207 | Wet-lab validated |
| fca-miR-193b-3p | AACUGGCCCACAAAGUCCCGCU | YP00205062 | Wet-lab validated |
| fca-miR-195-5p | UAGCAGCACAGAAAUAUUGGCA | YP00205969 | Wet-lab validated |
| fca-miR-199-3p | ACAGUAGUCUGCACAUUGGUUA | YP00204536 | Wet-lab validated |
| fca-miR-199a-5p | CCCAGUGUUCAGACUACCUGUU | YP02107249 | In silico validated |
| fca-miR-19b-3p | UGUGCAAAUCCAUGCAAAACUGA | YP00204450 | Wet-lab validated |
| fca-miR-200a-3p | UAACACUGUCUGGUAACGAUGU | YP00204707 | Wet-lab validated |
| fca-miR-200b-3p | UAAUACUGCCUGGUAAUGAUGA | YP00206071 | Wet-lab validated |
| fca-miR-200c-3p | UAAUACUGCCGGGUAAUGAUGGA | YP00204482 | Wet-lab validated |
| fca-miR-204-5p | UUCCCUUUGUCAUCCUAUGCCU | YP00206072 | Wet-lab validated |
| fca-miR-205-5p | UCCUUCAUUCCACCGGAGUCUGU | YP00205958 | Wet-lab validated |
| fca-miR-20a-5p | UAAAGUGCUUAUAGUGCAGGUA | YP02102800 | In silico validated |
| fca-miR-20b-5p | CAAAGUGCUCAUAGUGCAGGUAG | YP00204755 | Wet-lab validated |
| fca-miR-210-3p | CUGUGCGUGUGACAGCGGCUGA | YP00204333 | Wet-lab validated |
| fca-miR-214-3p | ACAGCAGGCACAGACAGGCAGU | YP00204510 | Wet-lab validated |
| fca-miR-21-5p | UAGCUUAUCAGACUGAUGUUGA | YP00204230 | Wet-lab validated |
| fca-miR-22-3p | AAGCUGCCAGUUGAAGAACUGU | YP00204606 | Wet-lab validated |
| fca-miR-25-3p | CAUUGCACUUGUCUCGGUCUGA | YP00204361 | Wet-lab validated |
| fca-miR-26a-5p | UUCAAGUAAUCCAGGAUAGGCU | YP00206023 | Wet-lab validated |
| fca-miR-26b-5p | UUCAAGUAAUUCAGGAUAGGUU | YP00205953 | Wet-lab validated |
| fca-miR-27a-3p | UUCACAGUGGCUAAGUUCCGC | YP00206038 | Wet-lab validated |
| fca-miR-27b-3p | UUCACAGUGGCUAAGUUCUGC | YP00205915 | Wet-lab validated |
| fca-miR-29a-3p | UAGCACCAUCUGAAAUCGGUUA | YP00204698 | Wet-lab validated |
| fca-miR-29b-3p | UAGCACCAUUUGAAAUCAGU | YP02100592 | In silico validated |
| fca-miR-31-5p | AGGCAAGAUGCUGGCAUAGCUGU | YP02119121 | In silico validated |
| fca-miR-328-3p | CUGGCCCUCUCUGCCCUUCCGU | YP00204364 | Wet-lab validated |
| fca-miR-340-5p | UUAUAAAGCAAUGAGACUGAUU | YP00206068 | Wet-lab validated |
| fca-miR-424-5p | CAGCAGCAAUUCAUGUUUUGA | YP02105705 | In silico validated |
| fca-miR-485-5p | AGAGGCUGGCCGUGAUGAAUUCG | YP02115752 | In silico validated |
| fca-miR-495-3p | AAACAAACAUGGUGCACUUCUU | YP00206015 | Wet-lab validated |
| fca-miR-497-5p | CAGCAGCACACUGUGGUUUGUA | YP00205164 | Wet-lab validated |
| fca-miR-7-5p | UGGAAGACUAGUGAUUUUGUUGUU | YP02102959 | In silico validated |
| hsa-miR-93-5p | CAAAGUGCUGUUCGUGCAGGUAG | YP00204715 | Wet-lab validated |
| hsa-miR-96-5p | UUUGGCACUAGCACAUUUUUGCU | YP00204417 | Wet-lab validated |
| hsa-miR-98-5p | UGAGGUAGUAAGUUGUAUUGUU | YP00204640 | Wet-lab validated |
| fca-miR-103-3p | AGCAGCAUUGUACAGGGCUAUGA | YP00204063 | Wet-lab validated |
| fca-miR-126-3p | UCGUACCGUGAGUAAUAAUGCG | YP00204227 | Wet-lab validated |
| fca-miR-134-5p | UGUGACUGGUUGACCAGAGGGG | YP00205989 | Wet-lab validated |
| fca-miR-135b-5p | UAUGGCUUUUCAUUCCUAUGUGA | YP00204130 | Wet-lab validated |
| fca-miR-150-5p | UCUCCCAACCCUUGUACCAGUG | YP00204660 | Wet-lab validated |
| fca-miR-151-5p | UCGAGGAGCUCACAGUCUAGU | YP00204007 | Wet-lab validated |
| fca-miR-153-3p | UUGCAUAGUCACAAAAGUGAUC | YP00204338 | Wet-lab validated |
| fca-miR-183-5p | UAUGGCACUGGUAGAAUUCACU | YP00206030 | Wet-lab validated |
| fca-miR-188-3p | CUCCCACAUGCAGGGUUUGCA | YP02119296 | Wet-lab validated |
| fca-miR-18a-3p | ACUGCCCUAAGUGCUCCUUCUGG | YP00204523 | Wet-lab validated |
| fca-miR-192-3p | CUGCCAAUUCCAUAGGUCACAG | YP00204272 | Wet-lab validated |
| fca-miR-194-5p | UGUAACAGCAACUCCAUGUGGA | YP00204080 | Wet-lab validated |
| fca-miR-197-3p | UUCACCACCUUCUCCACCCAGC | YP00204380 | Wet-lab validated |
| fca-miR-199b-5p | CCCAGUGUUUAGACUAUCUGUUC | YP00204152 | Wet-lab validated |
| fca-miR-216a-5p | UAAUCUCAGCUGGCAACUGUGA | YP00204167 | Wet-lab validated |
| fca-miR-218-5p | UUGUGCUUGAUCUAACCAUGU | YP00206034 | Wet-lab validated |
| fca-miR-22-5p | AGUUCUUCAGUGGCAAGCUUUA | YP00204255 | Wet-lab validated |
| fca-miR-30b-5p | UGUAAACAUCCUACACUCAGCU | YP00204765 | Wet-lab validated |
| fca-miR-335-3p | UUUUUCAUUAUUGCUCCUGACC | YP00205613 | Wet-lab validated |
| fca-miR-342-3p | UCUCACACAGAAAUCGCACCCGU | YP00205625 | Wet-lab validated |
| fca-miR-361-5p | UUAUCAGAAUCUCCAGGGGUAC | YP00206054 | Wet-lab validated |
| fca-miR-365-3p | UAAUGCCCCUAAAAAUCCUUAU | YP00204622 | Wet-lab validated |
| fca-miR-378-5p | CUCCUGACUCCAGGUCCUGUGU | YP00204347 | Wet-lab validated |
| fca-miR-382-5p | GAAGUUGUUCGUGGUGGAUUCG | YP00204169 | Wet-lab validated |

**Table S2: Specific miRNA targets investigated in Cohort 1 with fold change and p-values included.**

| **miRNA ID** | **2^-^ ^∆ Ct^**  **Margin Tissue** | **2^-^ ^∆ Ct^**  **Cancer Tissue** | **Fold Change** | **P-value** |
| --- | --- | --- | --- | --- |
| fca-let-7a-5p | 20.680176 | 21.065506 | 1.02 | 0.81 |
| fca-let-7c-5p | 8.714190 | 8.065817 | 0.93 | 0.55 |
| fca-let-7e-5p | 3.823110 | 3.427913 | 0.90 | 0.48 |
| fca-let-7f-5p | 2.320067 | 1.845985 | 0.80 | 0.19 |
| fca-let-7g-5p | 5.228824 | 4.628423 | 0.89 | 0.52 |
| fca-let-7i-5p | 4.540013 | 4.898366 | 1.08 | 0.44 |
| fca-miR-100-5p | 1.179452 | 0.738081 | 0.63 | 0.10 |
| fca-miR-10a-5p | 1.108383 | 0.648267 | 0.58 | 0.16 |
| fca-miR-10b-5p | 2.738805 | 1.496395 | 0.55 | 0.12 |
| fca-miR-125b-1-3p | 0.030162 | 0.028204 | 0.94 | 0.88 |
| fca-miR-125b-5p | 22.992581 | 21.490552 | 0.93 | 0.69 |
| fca-miR-128-3p | 0.349505 | 0.298843 | 0.86 | 0.48 |
| fca-miR-129-5p | 0.004357 | 0.004350 | 1.00 | 0.30 |
| fca-miR-130a-3p | 0.961598 | 1.056469 | 1.10 | 0.40 |
| fca-miR-130b-3p | 0.081728 | 0.111543 | 1.36 | 0.10 |
| fca-miR-132-3p | 0.015390 | 0.017266 | 1.12 | 0.71 |
| fca-miR-1-3p | 3.012657 | 1.217997 | 0.40 | 0.15 |
| fca-miR-140-5p | 0.511894 | 0.543198 | 1.06 | 0.45 |
| fca-miR-148a-3p | 3.371654 | 2.904844 | 0.86 | 0.66 |
| fca-miR-152-3p | 0.903049 | 0.787609 | 0.87 | 0.60 |
| fca-miR-155-5p | 0.440446 | 0.427436 | 0.97 | 0.67 |
| fca-miR-15a-5p | 3.257977 | 3.469820 | 1.07 | 0.34 |
| fca-miR-15b-5p | 0.461271 | 0.758216 | 1.64 | 0.03 |
| fca-miR-16-5p | 14.526815 | 14.649918 | 1.01 | 0.91 |
| fca-miR-17-5p | 2.749304 | 3.734437 | 1.36 | 0.09 |
| fca-miR-181a-5p | 1.347968 | 1.379266 | 1.02 | 0.91 |
| fca-miR-181b-5p | 0.296038 | 0.301149 | 1.02 | 0.82 |
| fca-miR-181c-5p | 0.021712 | 0.030256 | 1.39 | 0.19 |
| fca-miR-181d-5p | 0.010284 | 0.013345 | 1.30 | 0.26 |
| fca-miR-182 | 0.025016 | 0.156002 | 6.24 | 0.10 |
| fca-miR-186-5p | 0.597565 | 0.618648 | 1.04 | 0.71 |
| fca-miR-18a-5p | 0.135745 | 0.201961 | 1.49 | 0.16 |
| fca-miR-193b-3p | 2.428973 | 1.390324 | 0.57 | 0.19 |
| fca-miR-195-5p | 4.167079 | 4.003136 | 0.96 | 0.82 |
| fca-miR-199-3p | 7.963080 | 7.218308 | 0.91 | 0.96 |
| fca-miR-199a-5p | 5.439057 | 5.440204 | 1.00 | 0.69 |
| fca-miR-19b-3p | 7.709496 | 10.512404 | 1.36 | 0.10 |
| fca-miR-200a-3p | 0.231764 | 2.077316 | 8.96 | 0.16 |
| fca-miR-200b-3p | 0.396485 | 2.512390 | 6.34 | 0.14 |
| fca-miR-200c-3p | 0.779849 | 4.916837 | 6.30 | 0.08 |
| fca-miR-204-5p | 0.370731 | 0.149145 | 0.40 | 0.32 |
| fca-miR-205-5p | 0.942586 | 5.408283 | 5.74 | 0.52 |
| fca-miR-20a-5p | 2.329203 | 3.198489 | 1.37 | 0.06 |
| fca-miR-20b-5p | 0.007570 | 0.007794 | 1.03 | 0.89 |
| fca-miR-210-3p | 0.174695 | 0.568109 | 3.25 | 0.09 |
| fca-miR-214-3p | 1.489818 | 1.368979 | 0.92 | 0.86 |
| fca-miR-21-5p | 35.860570 | 75.073959 | 2.09 | 0.28 |
| fca-miR-22-3p | 12.810781 | 5.866218 | 0.46 | 0.08 |
| fca-miR-25-3p | 1.440946 | 2.005735 | 1.39 | 0.26 |
| fca-miR-26a-5p | 16.675944 | 12.089399 | 0.72 | 0.16 |
| fca-miR-26b-5p | 5.868194 | 4.892564 | 0.83 | 0.58 |
| fca-miR-27a-3p | 17.567860 | 16.442647 | 0.94 | 0.88 |
| fca-miR-27b-3p | 14.407884 | 13.954327 | 0.97 | 0.87 |
| fca-miR-29a-3p | 7.839527 | 6.293797 | 0.80 | 0.84 |
| fca-miR-29b-3p | 3.074553 | 1.891788 | 0.62 | 0.32 |
| fca-miR-31-5p | 0.091512 | 0.352282 | 3.85 | 0.08 |
| fca-miR-328-3p | 0.183084 | 0.139904 | 0.76 | 0.62 |
| fca-miR-340-5p | 0.125076 | 0.171504 | 1.37 | 0.09 |
| fca-miR-424-5p | 0.690803 | 1.264547 | 1.83 | 0.18 |
| fca-miR-485-5p | 0.002518 | 0.001533 | 0.61 | 0.97 |
| fca-miR-495-3p | 0.028020 | 0.028228 | 1.01 | 0.74 |
| fca-miR-497-5p | 2.415206 | 2.669428 | 1.11 | 0.59 |
| fca-miR-7-5p | 0.066302 | 0.051281 | 0.77 | 0.28 |
| hsa-miR-93-5p | 1.475492 | 1.945135 | 1.32 | 0.21 |
| hsa-miR-96-5p | 0.045722 | 0.421432 | 9.22 | 0.06 |
| hsa-miR-98-5p | 0.278778 | 0.250514 | 0.90 | 0.36 |
| fca-miR-103-3p | 2.752641 | 2.545291 | 0.92 | 0.61 |
| fca-miR-126-3p | 11.522777 | 5.747405 | 0.50 | 0.14 |
| fca-miR-134-5p | 0.001456 | 0.001351 | 0.93 | 0.67 |
| fca-miR-135b-5p | 0.008762 | 0.030336 | 3.46 | 0.94 |
| fca-miR-150-5p | 1.656188 | 1.658525 | 1.00 | 0.51 |
| fca-miR-151-5p | 1.628966 | 1.391924 | 0.85 | 0.15 |
| fca-miR-153-3p | 0.014316 | 0.007681 | 0.54 | 0.15 |
| fca-miR-183-5p | 0.020130 | 0.098616 | 4.90 | 0.12 |
| fca-miR-188-3p | 0.005680 | 0.006614 | 1.16 | 0.58 |
| fca-miR-18a-3p | 0.027065 | 0.042280 | 1.56 | 0.19 |
| fca-miR-192-3p | 0.001214 | 0.000865 | 0.71 | 0.39 |
| fca-miR-194-5p | 0.034461 | 0.038336 | 1.11 | 0.66 |
| fca-miR-197-3p | 0.381133 | 0.314423 | 0.82 | 0.29 |
| fca-miR-199b-5p | 3.741110 | 2.777007 | 0.74 | 0.48 |
| fca-miR-216a-5p | 0.004528 | 0.002620 | 0.58 | 0.96 |
| fca-miR-218-5p | 0.455056 | 0.441459 | 0.97 | 0.80 |
| fca-miR-22-5p | 0.494296 | 0.230622 | 0.47 | 0.12 |
| fca-miR-30b-5p | 4.680740 | 4.284719 | 0.92 | 0.35 |
| fca-miR-335-3p | 0.017433 | 0.024550 | 1.41 | 0.46 |
| fca-miR-342-3p | 0.560792 | 0.772882 | 1.38 | 0.38 |
| fca-miR-361-5p | 1.006025 | 0.840594 | 0.84 | 0.20 |
| fca-miR-365-3p | 2.428788 | 1.254585 | 0.52 | 0.07 |
| fca-miR-378-5p | 0.548581 | 0.207848 | 0.38 | 0.06 |
| fca-miR-382-5p | 0.012427 | 0.012641 | 1.02 | 0.65 |

**Figure S2: Dot plots showing raw Ct data of each miRNA target investigated in tumours and tumour margins from Cohort 1 (n=10). The raw data shows individual variation within the groups.**

**
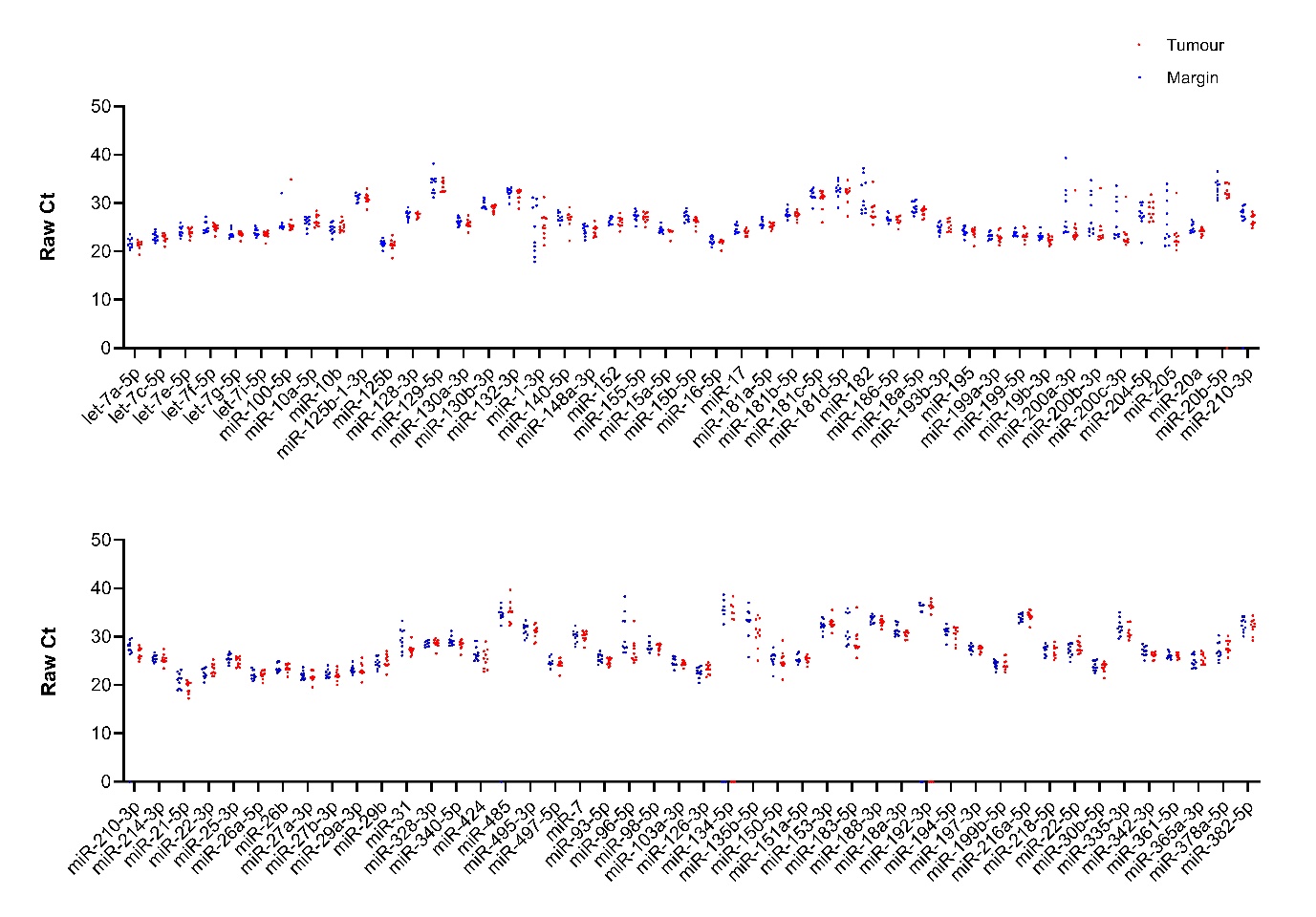
**

**Figure S3: Uncropped western blots are included in Figure 2 with corresponding Ponceau stain showing equal protein loading.**

The western blots were cut according to the molecular weight ladder and incubated separately due to limitations in sample volume. The Odyssey CLx Imaging system by Licor Biosciences automatically adjusts exposure for optimum detection as it analyses the membrane.


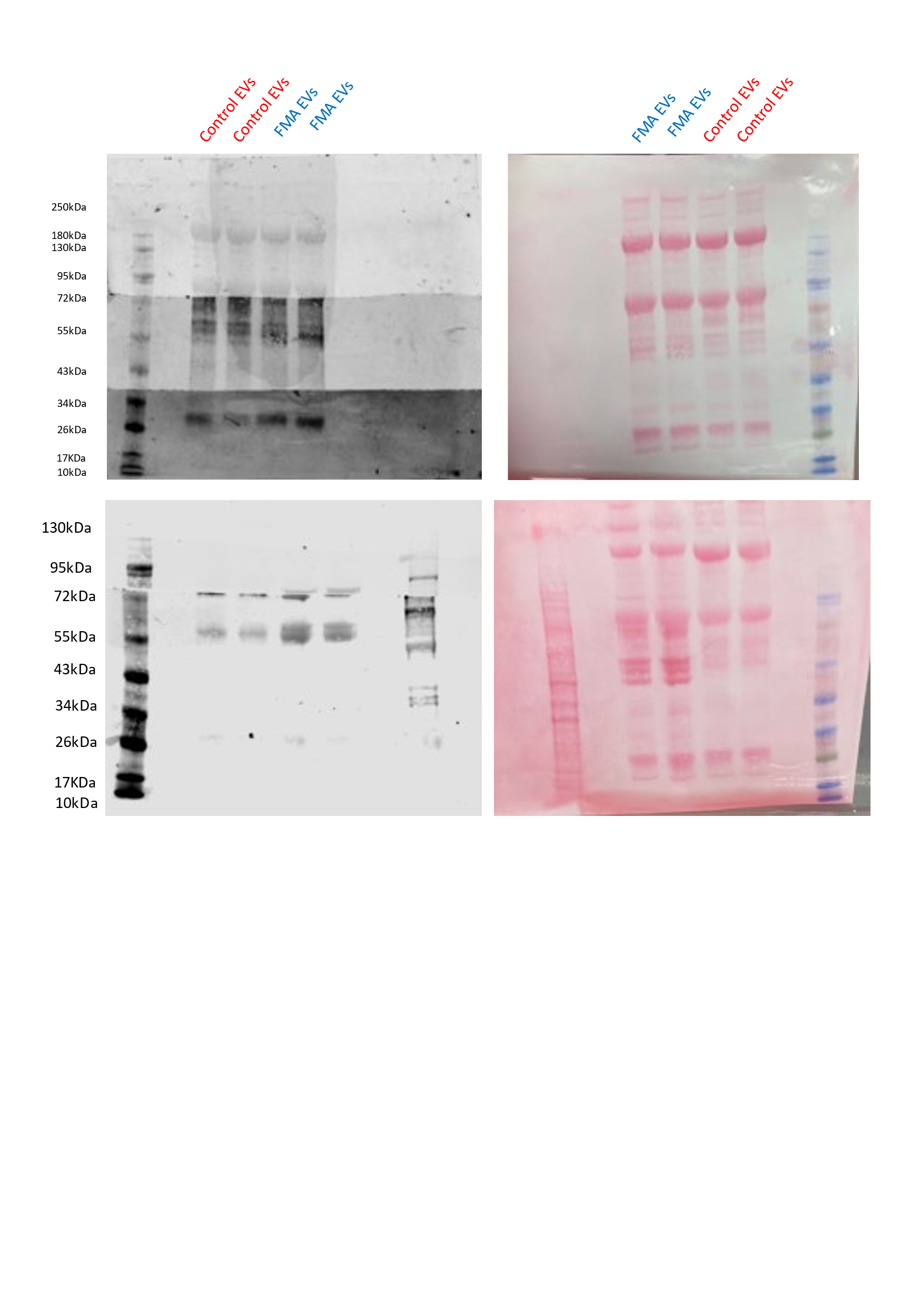


**Figure S4:** **There is no significant difference in miR-15b and miR-20a expression in FMA tumour tissue, margins or circulating EVs regardless of tumour grade.**

Matched tumour tissue (n=10) and tumour margins (n=10) isolated from felines with FMA were subjected to RT-qPCR for 8 miRNAs of interest. miRNA expression was divided based on tumour grade. Following the analysis of distribution using a Shapiro-Wilk test, miRNA expression was compared between groups using a one-way ANOVA. There was no significant difference in miR-20a or miR-15b expression in FFPE tumour tissue or FFPE tumour margins in feline patients with tumour grade. A plasma sample was also collected from each feline with FMA (n=10). Isolated EVs were subjected to RT-qPCR for 8 miRNAs of interest. Data was normalised by EV number ^31^. miR-20a and miR-15b showed no significant difference in expression regardless of tumour grade.


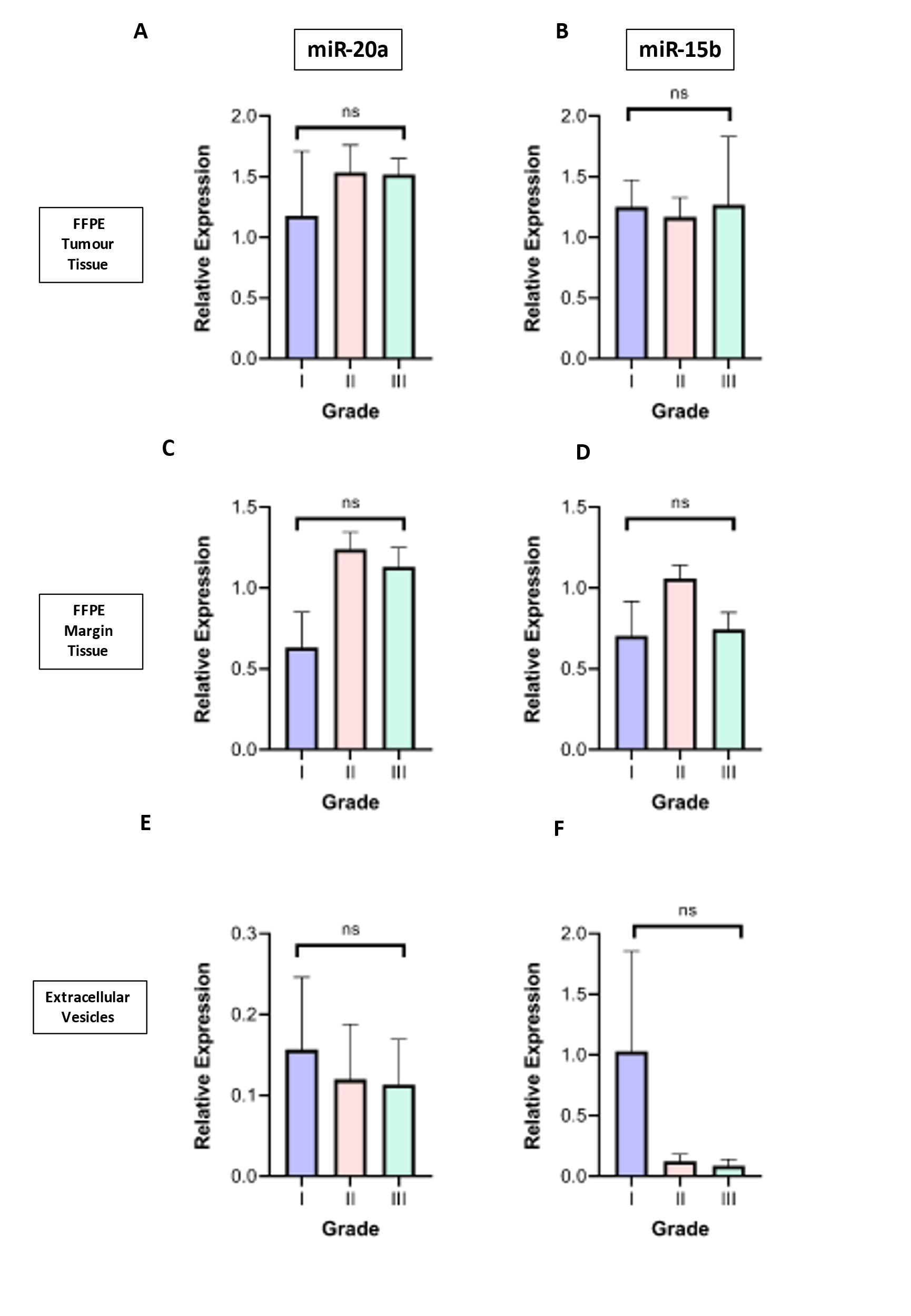


**Figure S5:** **There is no significant difference in miR-15b and miR-20a expression in FMA tumour tissue or tumour margins regardless of lymphatic invasion status. miR-20a expression is significantly increased in is reflected by significantly decreased expression in plasma EVs from patients with FMA compared to controls.** Matched tumour tissue (n=10) and tumour margins (n=10) isolated from felines with FMA were subjected to RT-qPCR for 8 miRNAs of interest. miRNA expression was divided based on lymphatic invasion status. Following analysis of distribution using a Shapiro-Wilk test, miRNA expression was compared between groups using an unpaired t-test. There was no significant difference in miR-20a or miR-15b expression in FFPE tumour tissue or FFPE tumour margins in feline patients with or without lymphatic invasion. A plasma sample was also collected from each feline with FMA (n=10). Isolated EVs were subjected to RT-qPCR for 8 miRNAs of interest. Data was normalised by EV number ^31^. miR-20a was significantly increased in feline patients with lymphatic invasion compared to controls, while miR-15b showed no significant difference in expression regardless of lymphatic invasion status.


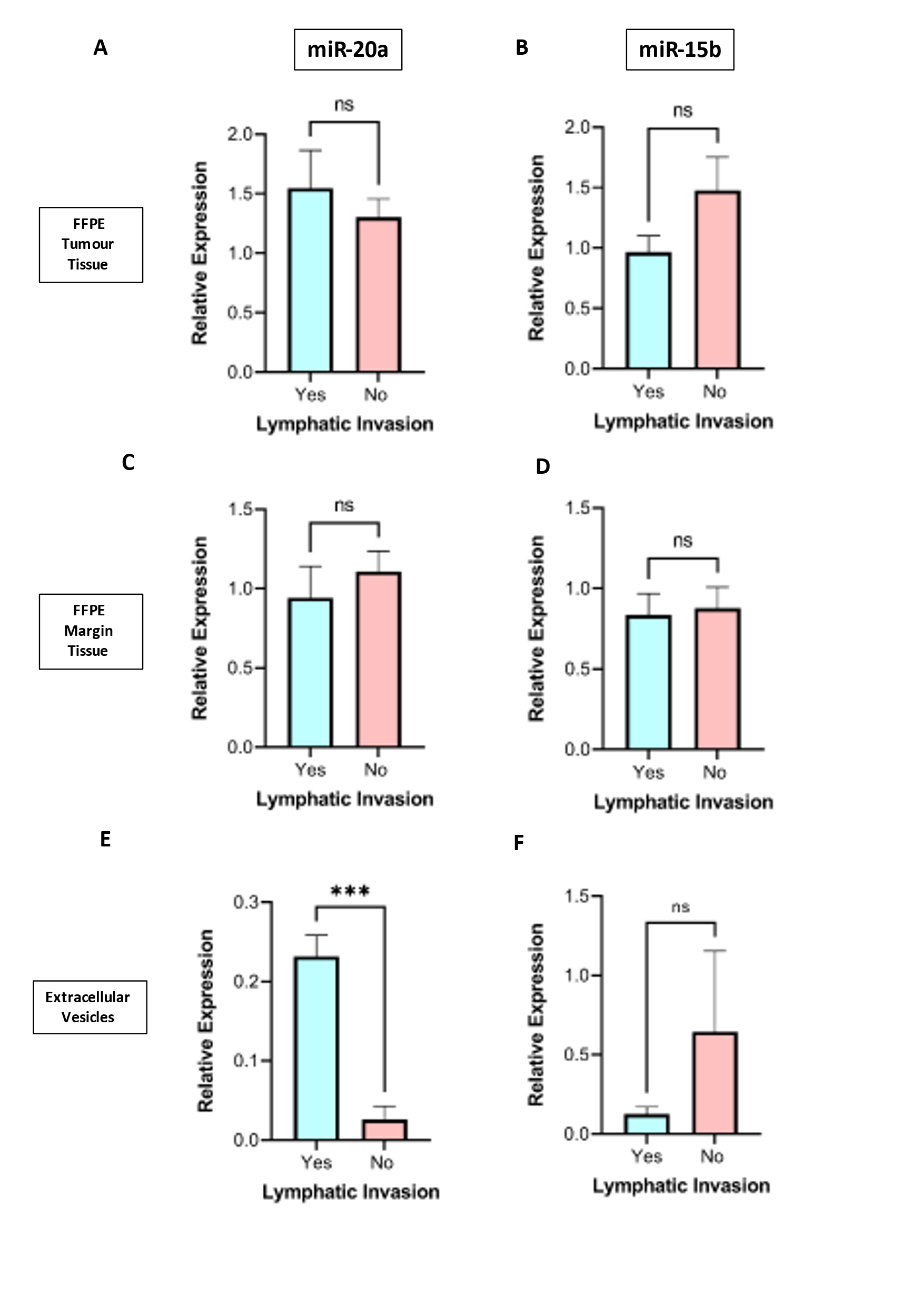


**Table S3: Comparison of the levels of protein targets of miR-20a and miR-15b based on tumour grade and lymphatic invasion.**

The relative quantity of proteins was compared by normality testing of data using a Shapiro-Wilk test. For the analysis of tumour grade, an ANOVA test was used to compare the relative quantity of proteins among different grades of tumour. For the analysis of lymphatic invasion, an unpaired t-test was used to compare the relative quantity of proteins between patients presenting with and without lymphatic invasion.

| **Protein Identification**  (Gene Name) | **Comparison of Tumour Grade (I, II, III)**  (p-value) | **Comparison of Lymphatic Invasion (Yes/No)**  (p-value) |
| --- | --- | --- |
| TPM4 | 0.169 | 0.159 |
| YWHAZ | 0.100 | 0.344 |
| ITGA2 | 0.001** | 0.680 |
| ARHGDIA | 0.685 | 0.090 |
| CPNE1 | 0.311 | 0.148 |
| ITGA6 | 0.407 | 0.390 |

**Figure S6: Comparative analysis of the number of proteins identified by Data Dependent Acquisition (DDA) and Data Independent Acquisition (DIA). Ninety percent of proteins identified by DDA were also identified by DIA, confirming the reproducibility of DIA data for mass spectrometry analysis of feline EVs.**


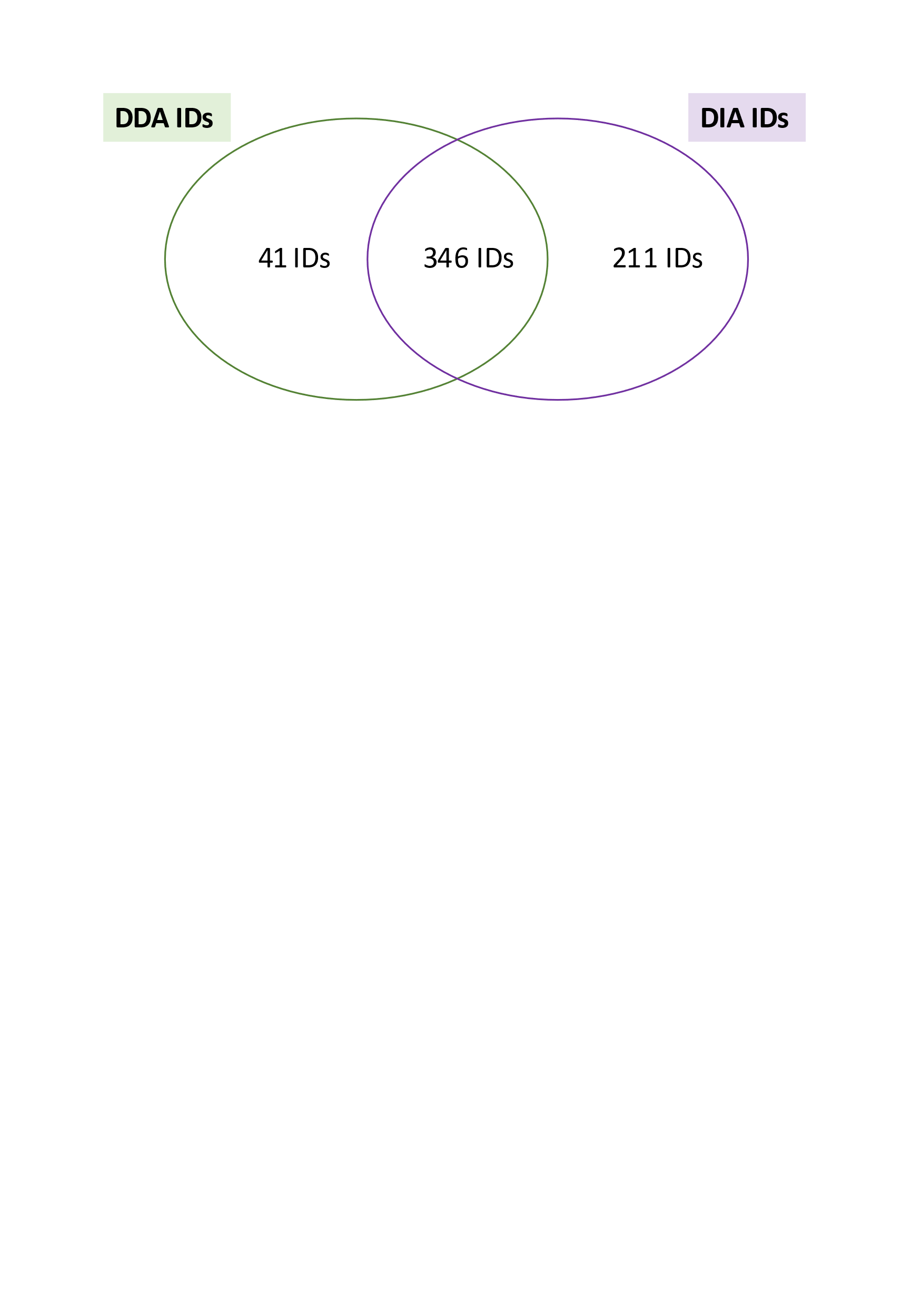


**Figure S7: Haematoxylin and Eosin stains of a grade 2 tubular adenocarcinoma sections with respective margins**

FMA tumours were placed in 10% neutral buffered formalin for a minimum of 20 hours. The tissues were then processed and embedded in paraffin-wax. Tissue blocks were sectioned at 4 µm. and stained with haematoxylin and eosin for histological grading ^18^. Representative images of haematoxylin and eosin stains of a grade 2 tubular adenocarcinoma sections with respective margins**.** **A:** Haematoxylin and eosin stain of tumour tissue, magnification: 1.25X, scale bar: 2mm (s: tumour stroma, t: tumour, n: normal tissue) **B:** Haematoxylin and eosin stain of margin tissue, magnification: 1.25X, scale bar: 2mm (a: adipose tissue (normal), d: mammary ducts)


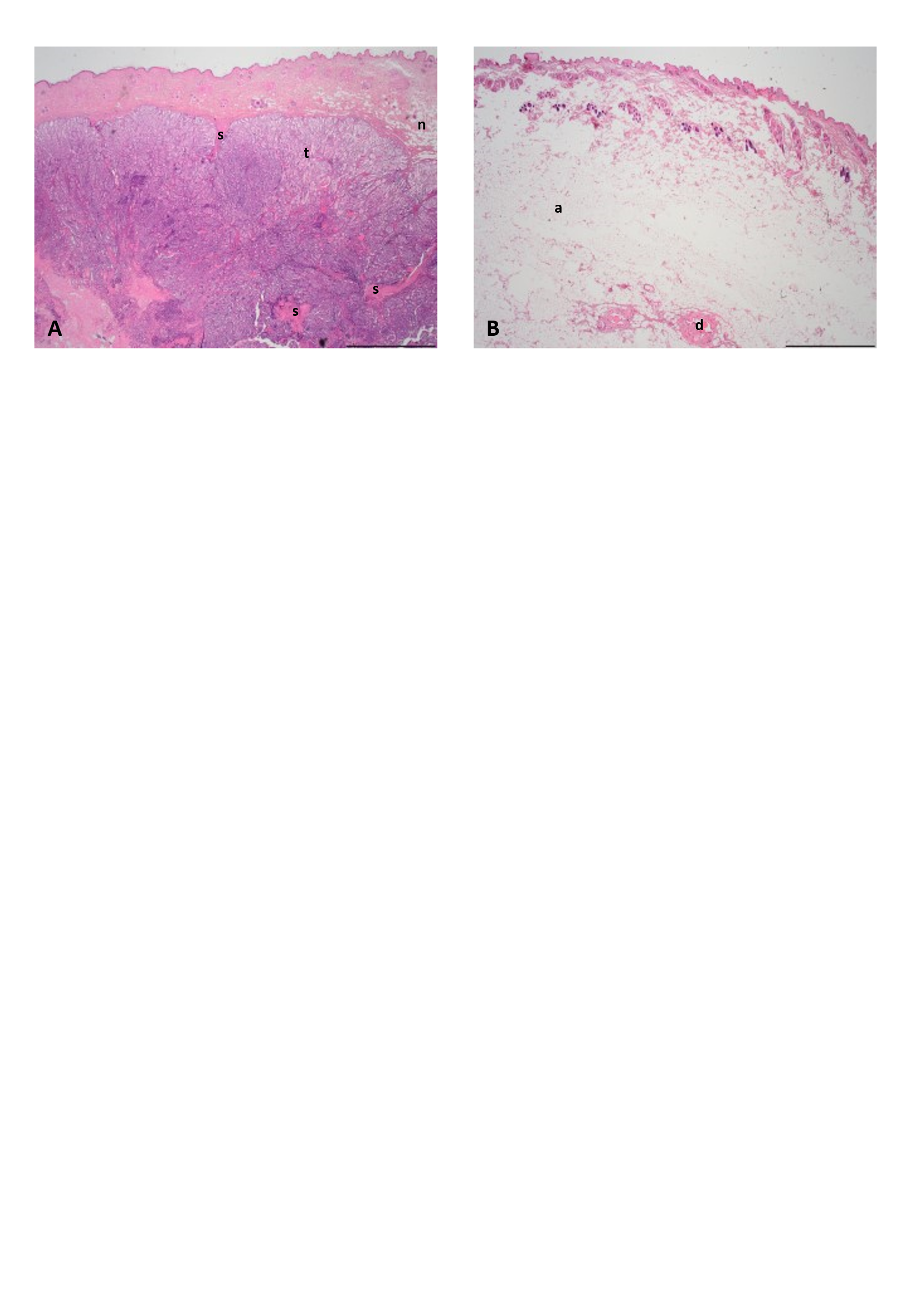

Supplement: Supplementary file 1 — Supplementary Information. [file 41598_2023_36110_MOESM1_ESM.docx]
